# Projeções de Mortalidade por Doença Isquêmica do Coração, Acidente Vascular Cerebral e Doença Arterial Periférica no Brasil até 2040: Uma Abordagem de Modelagem Bayesiana

**DOI:** 10.36660/abc.20250189

**Published:** 2025-12-18

**Authors:** Pedro Rafael Vieira de Oliveira Salerno, Antoinette Cotton, Bruno R. Nascimento, Zhuo Chen, Gabriel Tensol Rodrigues Pereira, Alexandre A. Abizaid, Luis Augusto Palma Dallan, Pedro Rafael Salerno, Sadeer Al-Kindi, Fanny Petermann-Rocha, Salil V. Deo

**Affiliations:** 1 Icahn School of Medicine at Mt. Sinai Queens NY EUA Department of Medicine, NYC Health + Hospitals/Elmhurst, Icahn School of Medicine at Mt. Sinai, Queens, NY – EUA; 2 University Hospitals of Cleveland Cleveland Ohio EUA University Hospitals of Cleveland, Cleveland, Ohio – EUA; 3 Case Western Reserve University Cleveland EUA Case Western Reserve University, Cleveland – EUA; 4 Universidade Federal de Minas Gerais Belo Horizonte MG Brasil Universidade Federal de Minas Gerais, Belo Horizonte, MG – Brasil; 5 Hospital Israelita Albert Einstein São Paulo SP Brasil Hospital Israelita Albert Einstein, São Paulo, SP – Brasil; 6 Instituto do Coração do Hospital das Clínicas Faculdade de Medicina Universidade de São Paulo São Paulo SP Brasil Instituto do Coração do Hospital das Clínicas da Faculdade de Medicina da Universidade de São Paulo, São Paulo, SP – Brasil; 7 Universidade Católica de Pernambuco Recife PE Brasil Universidade Católica de Pernambuco, Recife, PE – Brasil; 8 Center for Health and Nature Houston Methodist Houston EUA Center for Health and Nature Houston Methodist, Houston – EUA; 9 Universidad Diego Portales Santiago Chile Universidad Diego Portales, Santiago – Chile; 10 Louis Stokes VA Medical Center Cleveland Ohio EUA Louis Stokes VA Medical Center, Cleveland, Ohio – EUA; 11 University of Glasgow Glasgow Inglaterra University of Glasgow, Glasgow – Inglaterra

**Keywords:** Isquemia Miocárdica, Acidente Vascular Cerebral, Doença Arterial Periférica, Mortalidade, Brasil

## Abstract

**Fundamento:**

As doenças cardiovasculares ateroscleróticas — especialmente a doença isquêmica do coração (DIC), o acidente vascular cerebral (AVC) e a doença arterial periférica (DAP) — são a principal causa de mortalidade cardiovascular no Brasil.

**Objetivos:**

Projetar as tendências de mortalidade por DIC, AVC e DAP no Brasil até 2040.

**Métodos:**

As contagens anuais de óbitos (1990-2021) por DIC, AVC e DAP entre indivíduos de 40 a 79 anos foram obtidas a partir do estudo Carga Global de Doenças (*Global Burden of Disease*, GBD) de 2021. Utilizaram-se estimativas populacionais de meio de ano tanto para o período observado (1990-2021) quanto para o projetado (2022-2040). Foram calculadas as taxas de mortalidade brutas e padronizadas por idade. Modelos bayesianos idade-período-coorte foram aplicados para projetar as taxas de mortalidade de 2022 a 2040. Foram computadas as variações percentuais relativas e as estimativas anuais de variação percentual (EAVPs). As projeções (por 100.000 habitantes) são apresentadas como medianas com intervalos de incerteza (IIs) de 95%, e as EAVPs incluem intervalos de confiança (ICs) de 95% derivados por *bootstrap*.

**Resultados:**

Entre 1990 e 2040, estima-se que a população brasileira de 40 a 79 anos aumente em 237,82%. A taxa de mortalidade padronizada por idade para DIC deverá apresentar uma redução de 14,16% [de 118,61 em 2021 para 101,82 em 2040 (II 95%, 0,36-203,27)] (EAVP: -0,83% [IC 95%, -0,84 a -0,83]); e, para AVC, uma redução de 17,36% [de 84,58 para 69,90 (II 95%, 0-152,48)] (EAVP: -1,07% [IC 95%, -1,10 a -1,04]). Em contraste, projeta-se que a mortalidade por DAP aumente em 10,99% [de 1,82 para 2,02 (II 95%, 0-5,03)] (EAVP: 0,45% [IC 95%, 0,30-0,59]). Adicionalmente, as taxas de mortalidade padronizadas por idade, específicas por sexo, mostraram variações consideráveis. Para a DIC, projeta-se uma redução de 25,31% entre homens (EAVP, -1,56% [IC 95%, -1,57 a -1,55]), enquanto entre mulheres deverá haver um aumento de 4,12% (EAVP: 0,14% [IC 95%, 0,13-0,16]). A mortalidade por AVC deverá reduzir em 30,00% entre homens (EAVP, -1,94% [IC 95%: -1,96 a -1,91]) e em 4,52% entre mulheres (EAVP: -0,33% [IC 95%, -0,37 a -0,29]). Em contrapartida, a mortalidade por DAP deverá crescer 14,64% entre homens (EAVP, 0,55% [IC 95%: 0,38-0,71]) e 21,92% entre mulheres (EAVP: 0,91% [IC 95%: 0,78-1,02]).

**Conclusão:**

Embora se preveja redução nas taxas de mortalidade por DIC e AVC, espera-se um aumento na mortalidade por DAP — especialmente entre as mulheres —, o que evidencia a necessidade urgente de intervenções em saúde pública específicas por sexo e por tipo de doença.

## Graphical abstract

**Figura Central:**
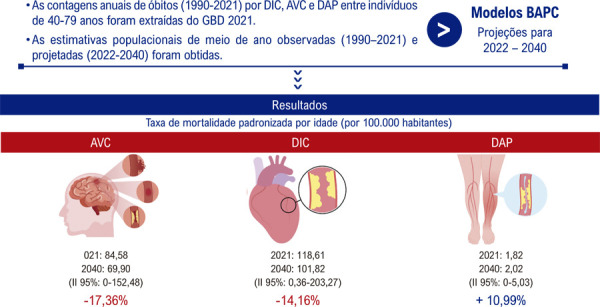
Projeções de Mortalidade por Doença Isquêmica do Coração, Acidente Vascular Cerebral e Doença Arterial Periférica no Brasil até 2040: Uma Abordagem de Modelagem Bayesiana

Taxas de mortalidade padronizadas por idade projetadas (por 100.000 habitantes) para AVC, DIC e DAP no Brasil. As projeções foram derivadas por meio de modelagem bayesiana, com base em dados observados de mortalidade de 1990 a 2021 e em estimativas populacionais de meio de ano para o período de 2022 a 2040. Os valores para 2021 e 2040 são apresentados, juntamente com seus respectivos II 95%. Os resultados indicam reduções projetadas nas taxas de mortalidade por AVC e DIC de 17,36% e 14,16%, respectivamente, enquanto se espera um aumento de 10,99% na mortalidade por DAP. As taxas específicas de mortalidade para 2021 e 2040 estão apresentadas abaixo de cada condição. AVC: acidente vascular cerebral; BAPC: modelo bayesiano idade-período-coorte; GBD: Global Burden of Disease; DAP: doença arterial periférica; DIC: doença isquêmica do coração; II: intervalo de incerteza.

## Introdução

As doenças cardiovasculares (DCVs) ateroscleróticas englobam 3 condições principais: doença isquêmica do coração (DIC), acidente vascular cerebral (AVC) e doença arterial periférica (DAP). Em 2013, DIC e AVC juntos foram responsáveis por 247,9 óbitos por 100.000 habitantes em todo o mundo.^1^ Por muitos anos, ambas têm figurado consistentemente entre as principais causas de mortalidade cardiovascular na maioria dos países. Em 2019, o estudo Carga Global de Doença (*Global Burden of Disease*, GBD) relatou que 113 milhões de adultos em todo o mundo eram afetados pela DAP, com 42% dos óbitos relacionados ocorrendo em países de baixa e média renda, incluindo o Brasil.^2^ Segundo o relatório Estatística Cardiovascular – Brasil 2023, as DCVs continuam sendo a principal causa de morte no país.^3^

Embora a taxa de mortalidade padronizada por idade por DCV tenha diminuído nas últimas décadas no Brasil, a prevalência dessas condições aumentou em 26%.^4,5^ As taxas de obesidade e dislipidemia permanecem elevadas e, de acordo com o relatório de 2024 da Organização Mundial da Saúde (OMS), o Brasil figura entre os países com maior uso de tabaco. Além disso, estima-se que a mortalidade atribuível ao diabetes no país aumente em 144% nas próximas 2 décadas.^4,6^

Reconhecendo o impacto negativo das condições cardiovasculares na saúde da população, o Congresso Nacional Brasileiro promulgou, em 1990, a Lei nº 8.080, que instituiu o Sistema Único de Saúde (SUS), sistema público de saúde gratuito e universal. Atualmente, o SUS atende mais de 70% da população brasileira.^7^ Adicionalmente, o Ministério da Saúde implementou o Programa Saúde da Família em meados da década de 1990, seguido por estratégias de prevenção primária em 2002 e pela adoção de uma política de cobertura de medicamentos genéricos para o tratamento da hipertensão, diabetes e dislipidemia.^8^ Essas iniciativas contribuíram para melhorias na saúde da população. No entanto, à medida que as políticas públicas evoluem e as opções terapêuticas se expandem, a carga atribuível às DCVs e seus componentes pode se modificar. Assim, compreender a trajetória futura dessas condições é essencial para orientar estratégias nacionais de saúde.

Alguns países, como Estados Unidos, Japão e Reino Unido, já realizaram projeções sobre a saúde cardiovascular.^9,10^ Nos EUA e no Japão, projeta-se redução na mortalidade por DIC, enquanto a mortalidade por AVC deverá permanecer relativamente estável. No entanto, tais achados não podem ser generalizados para outros contextos, devido às variações na estrutura populacional, nas taxas de fecundidade, nos padrões de envelhecimento e nos fluxos migratórios. Como o Brasil está entre os países mais populosos do mundo, sua contribuição para as estimativas globais é significativa.

Assim, o presente estudo teve como objetivo projetar as taxas de mortalidade por DIC, AVC e DAP entre adultos brasileiros com idade entre 40 e 79 anos até 2040, com base em dados observados de 1990 a 2021.

## Métodos

### Fonte de dados

#### Mortalidade por DIC, AVC e DAP

A principal fonte de dados deste estudo foi o estudo GBD 2021.^11^ O GBD, um consórcio global com mais de 12.000 colaboradores, fornece dados padronizados sobre incapacidade e mortalidade atribuíveis a diversas condições de saúde e fatores de risco em 204 países e territórios.^12^ A atualização de 2021 oferece acesso público por meio da ferramenta de consulta da *Global Health Data Exchange* (GHDx) (http://ghdx.healthdata.org/gbd-results-tool).^11^ Como signatário das *Guidelines for Accurate and Transparent Health Estimates Reporting* da OMS, o GBD aplica um processamento padronizado de dados para garantir a comparabilidade entre os países.^12^

Utilizando o GHDx, extraímos as contagens anuais de óbitos atribuídos à DIC, AVC e DAP no Brasil em intervalos etários de 5 anos (40-44, 45-49, 50-54, 55-59, 60-64, 65-69, 70-74 e 75-79 anos), de 1990 a 2021. O GBD 2021 define DIC como um composto de infarto agudo do miocárdio, angina estável crônica, doença arterial coronariana e miocardiopatia isquêmica, e define DAP como índice tornozelo-braquial < 0,9.^13^ De acordo com o GBD, a fonte original dos dados de mortalidade foi o Sistema de Informações sobre Mortalidade (SIM), um banco de dados nacional de atestados de óbito curado e avaliado quanto à qualidade, gerenciado pelo Ministério da Saúde do Brasil.^14^ Os dados brutos foram processados utilizando o modelo *Cause of Death Ensemble* (CODEm), uma estrutura de regressão geoespacial bayesiana desenvolvida pelo *Institute for Health Metrics and Evaluation* (Seattle, WA).^13^ Informações adicionais sobre os dados do GBD 2021 utilizados neste estudo estão disponíveis no material suplementar.

#### Estimativas populacionais

Também obtivemos estimativas populacionais de meio de ano para os mesmos intervalos etários (40-44 a 75-79 anos) no período de 1990 a 2040, a partir do GBD 2021.^15,16^ Tais estimativas são oriundas do Instituto Brasileiro de Geografia e Estatística, responsável pelos censos nacionais no Brasil.^15^ Os dados foram extraídos para a população geral, bem como desagregados por sexo.

## Análise estatística

Inicialmente, calculamos as taxas de mortalidade anuais observadas, brutas e padronizadas por idade (por 100.000 habitantes), para DIC, AVC e DAP entre 1990 e 2021. As taxas padronizadas por idade e os respectivos intervalos de confiança (ICs) 95% foram calculados utilizando a população de meio de ano de 1990 como padrão, com os ICs estimados por meio do método delta.

Em seguida, aplicamos modelos bayesianos idade-período-coorte (IPC) aos dados observados (1990-2021) para projetar as taxas de mortalidade por DIC, AVC e DAP até 2040. Os modelos IPC permitem a modelagem simultânea dos efeitos de idade, período e coorte em uma única equação de regressão e podem acomodar relações não lineares.^17^ Utilizamos *priors* do tipo *random walk 2* com distribuição log-gama, e modelamos a variável desfecho (eventos de mortalidade) por meio de uma estrutura de regressão linear generalizada com função *log-link* e distribuição de Poisson.

Embora existam diversas formulações possíveis para os modelos IPC, estudos recentes de simulação demonstraram que os modelos bayesianos IPC oferecem melhor acurácia preditiva.^18^ As estimativas dos modelos ajustados foram obtidas utilizando a *integrated nested Laplace approximation*, uma abordagem computacionalmente eficiente, conhecida por produzir estimativas posteriores estáveis e confiáveis sem problemas de convergência, comparáveis às obtidas por simulações de Cadeia de Markov de Monte Carlo.^19^ Informações detalhadas sobre a especificação dos priors, metodologia de projeção e procedimentos de avaliação dos modelos estão disponíveis no material suplementar.

Os modelos foram ajustados para a população brasileira total e separadamente para homens e mulheres. Para cada grupo, as taxas de mortalidade foram projetadas para todos os intervalos etários de 5 anos definidos, e as taxas padronizadas por idade foram novamente indexadas à população de meio de ano de 1990. As taxas projetadas são apresentadas como medianas com intervalos de incerteza (IIs) de 95%. Também calculamos a variação percentual relativa e a estimativa anual de variação percentual (EAVP) entre o último ano observado (2021) e o último ano projetado (2040). As EAVPs foram estimadas por regressão linear generalizada com função log-link, e os ICs 95% foram obtidos por reamostragem *bootstrap*.

Todas as análises foram realizadas no R versão 4.3.0 (*R Foundation for Statistical Computing*, Viena, Áustria). Como o estudo utilizou exclusivamente dados públicos em nível populacional, foi isento de aprovação por Comitê de Ética em Pesquisa com Seres Humanos e não exigiu consentimento informado. Este estudo foi conduzido e reportado em conformidade com as diretrizes *Strengthening the Reporting of Observational Studies in Epidemiology*.

### Envolvimento de pacientes e do público

Pacientes e membros do público não participaram do delineamento, execução, relato ou plano de disseminação desta pesquisa, uma vez que tal participação não foi considerada apropriada ou viável.

## Resultados

Em 1990, o Brasil possuía 32.751.825 residentes com idade entre 40 e 79 anos, dos quais 51,7% eram mulheres. Entre 1990 e 2040, projeta-se que esse grupo etário aumente em 237,8%, acompanhado por um envelhecimento populacional substancial (Figura S1). A proporção de adultos com idade entre 75 e 79 anos deverá praticamente dobrar, enquanto a participação de faixas etárias mais jovens (p.ex., 40-44 anos) deverá diminuir.

**Figura 1:**
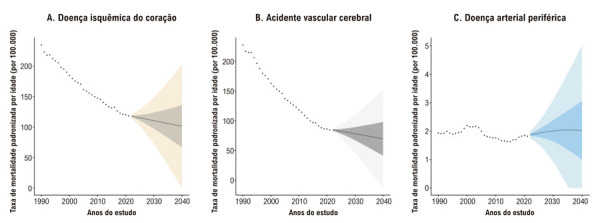
– Projeções das taxas de mortalidade padronizadas por idade no Brasil até 2040. Os painéis A, B e C apresentam, respectivamente, as taxas de mortalidade padronizadas por idade (por 100.000 habitantes) para DIC, AVC e DAP. As estimativas foram obtidas por meio de modelos bayesianos idade-período-coorte, com base em dados observados de 1990 a 2021. Os pontos pretos representam os valores observados (1990-2021), enquanto os gráficos em leque ilustram as estimativas projetadas para 2022-2040. As áreas sombreadas indicam os intervalos de incerteza: as faixas mais escuras representam o intervalo interquartil (25º ao 75º percentil) e as faixas mais claras representam o II 95% (2,5º ao 97,5º percentil). AVC: acidente vascular cerebral; DAP: doença arterial periférica; DIC: doença isquêmica do coração; II: intervalo de incerteza.

**Figura 2:**
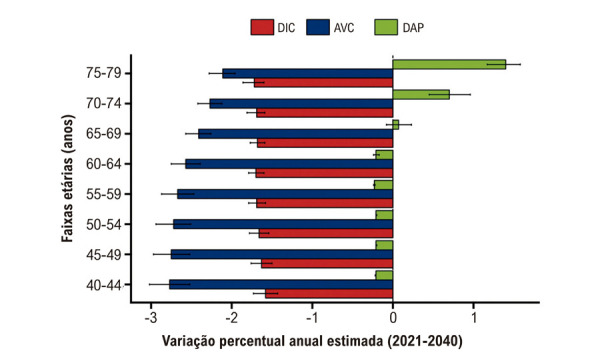
– Variação projetada na mortalidade por DIC, AVC e DAP no Brasil por faixa etária, 2021-2040. Esta figura apresenta a EAVP nas taxas de mortalidade (por 100.000 habitantes) por DIC, AVC e DAP em faixas etárias de 5 anos no Brasil, projetadas para o período de 2021 a 2040. As barras representam as estimativas pontuais, e as linhas de erro indicam os IC 95%. Os valores completos da EAVP por faixa etária e condição estão apresentados na Tabela 2. AVC: acidente vascular cerebral; EAVP: estimativa anual de variação percentual; DAP: doença arterial periférica; DIC: doença isquêmica do coração; IC: intervalo de confiança.

A taxa de mortalidade padronizada por idade por DIC apresentou uma redução substancial entre 1990 e 2021 (Tabela S1, Figura S2), e essa tendência deverá se manter até 2040 (Tabela 1, Figura 1). Embora se prevejam reduções em todas as faixas etárias, a maior queda é esperada entre indivíduos de 60 a 64 anos (Tabela 1, Figura S3, Figura 2).

**Tabela 1:** – Taxas de mortalidade projetadas (por 100.000 habitantes) por DIC, AVC e DAP no Brasil, por grupos etários de 5 anos (40-79 anos), para 2021 (observado), 2030 e 2040 (projetado)

		Ano	Taxa padronizada por idade	Faixa etária (anos)							
				40-44	45-49	50-54	55-59	60-64	65-69	70-74	75-79
**DIC**	Observado	2021	118,61 (117,90-119,32)	21,37 (20,68-22,09)	40,30 (39,27-41,36)	70,76 (69,33-72,23)	112,61 (110,70-114,54)	173,32 (170,74-175,94)	249,85 (246,35-253,40)	354,19 (349,37-359,08)	516,39 (509,22-523,65)
	Projetado	2030	110,76 (72,36-149,16)	19,88 (13,48-29,54)	37,16 (25,90-53,67)	62,84 (44,09-90,21)	99,91 (70,11-143,41)	155,50 (109,13-223,17)	232,25 (162,99-333,31)	347,29 (243,72-498,40)	506,23 (355,24-726,46)
		2040	101,82 (0,36-203,27)	19,04 (6,38-57,74)	35,59 (12,40-103,55)	60,20 (21,46-171,18)	93,99 (33,83-264,76)	141,99 (51,21-399,30)	210,94 (76,06-593,26)	309,77 (111,71-871,19)	453,55 (163,55-1275,56)
	Variação percentual relativa		-14,16%	-10,90%	-11,69%	-14,92%	-16,53%	-18,08%	-15,58%	-12,54%	-12,17%
	EAVP		-0,83 (-0,84a-0,83)	-1,58 (-1,73a-1,43)	-1,63 (-1,76a-1,5)	-1,66 (-1,78a-1,54)	-1,69 (-1,79a-1,58)	-1,7 (-1,79a-1,6)	-1,68 (-1,77a-1,59)	-1,69 (-1,81a-1,59)	-1,72 (-1,86a-1,6)
**AVC**	Observado	2021	84,58 (83,99-85,18)	15,24 (14,66-15,85)	27,11 (26,27-27,98)	44,48 (43,35-45,65)	67,22 (65,75-68,72)	106,10 (104,09-108,16)	170,44 (167,56-173,38)	280,48 (276,18-284,83)	467,18 (460,37-474,10)
	Projetado	2030	78,38 (46,18-110,59)	13,56 (8,62-21,37)	23,28 (15,19-35,71)	37,83 (24,84-57,66)	58,81 (38,63-89,63)	94,08 (61,80-143,37)	155,30 (102,02-236,67)	273,05 (179,36-416,09)	466,67 (306,54-711,13)
		2040	69,90 (0-152,48)	12,35 (3,44-44,46)	21,20 (6,13-73,40)	34,45 (10,18-116,68)	52,89 (15,77-177,51)	83,52 (24,96-279,82)	139,11 (41,57-466,15)	240,41 (71,84-805,53)	410,59 (122,69-1375,73)
	Variação percentual relativa		-17,36%	-18,96%	-21,80%	-22,55%	-21,32%	-21,28%	-18,38%	-14,29%	-12,11%
	EAVP		-1,07 (-1,10a-1,04)	-2,77 (-3,02a-2,52)	-2,75 (-2,97a-2,52)	-2,72 (-2,94a-2,51)	-2,67 (-2,87a-2,47)	-2,57 (-2,75a-2,39)	-2,41 (-2,57a-2,26)	-2,27 (-2,42a-2,12)	-2,11 (-2,28a-1,96)
**DAP**	Observado	2021	1,82 (1,73-1,91)	0,13 (0,09-0,20)	0,27 (0,20-0,38)	0,58 (0,46-0,73)	1,28 (1,09-1,50)	2,38 (2,10-2,71)	4,05 (3,62-4,53)	7,12 (6,46-7,85)	12,08 (11,03-13,24)
	Projetado	2030	2.01 (0,91, 3,10)	0,12 (0,06-0,24)	0,26 (0,14-0,48)	0,56 (0,31-0,98)	1,17 (0,64-2,01)	2,34 (1,29-4,00)	4,52 (2,50-7,72)	8,44 (4,68-14,42)	14,92 (8,27-25,49)
		2040	2,02 (0-5,03)	0,12 (0,02-0,63)	0,26 (0,05-1,24)	0,55 (0,11-2,51)	1,14 (0,23-5,06)	2,28 (0,47-9,94)	4,41 (0,90-19,10)	8,49 (1,74-36,74)	16,23 (3,33-70,24)
	Variação percentual relativa		+10,99%	-7,69%	-3,70%	-5,17%	-10,94%	-4,20%	+8,89%	+19,24%	+34,35%
	EAVP		0,45 (0,30-0,59)	-0,21 (-0,22a-0,21)	-0,21 (-0,21a-0,21)	-0,21 (-0,21a-0,21)	-0,23 (-0,24a-0,23)	-0,21 (-0,24a-0,21)	0,07 (-0,08-0,07)	0,70 (0,45-0,70)	1,4 (1,17-1,40)

As taxas padronizadas por idade e específicas por faixa etária são apresentadas como medianas com intervalo de incerteza (II) para as projeções de 2030 e 2040, e como taxas brutas com IC 95% para o ano observado (2021). Também são fornecidas a variação percentual relativa e a EAVP entre 2021 e 2040. DAP: doença arterial periférica; DIC: doença isquêmica do coração; EAVP: estimativa anual de variação percentual; IC: intervalo de confiança.

**Figura 3:**
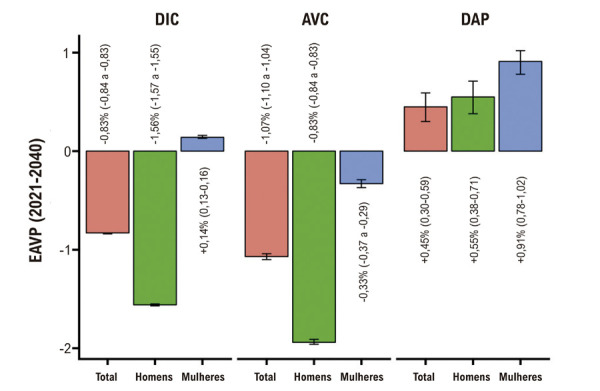
– Variação projetada na mortalidade por DIC, AVC e DAP no Brasil até 2040. Esta figura apresenta a EAVP nas taxas de mortalidade padronizadas por idade (por 100.000 habitantes) para DIC (Painel A), AVC (Painel B) e DAP (Painel C) entre 2021 e 2040. Os resultados são apresentados para a população total, bem como desagregados por sexo (homens e mulheres). AVC: acidente vascular periférico; DAP: doença arterial periférica; DIC: doença isquêmica do coração; EAVP: estimativa anual de variação.

**Figura 4:**
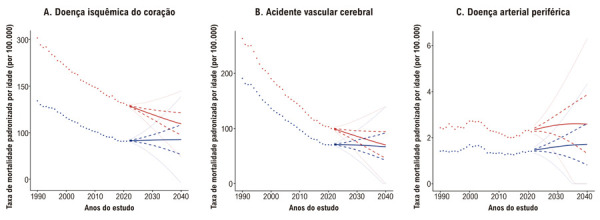
– Projeções das taxas de mortalidade padronizadas por idade em homens e mulheres no Brasil até 2040. Os painéis A, B e C apresentam as taxas de mortalidade padronizadas por idade (por 100.000 habitantes) para doença isquêmica do coração, acidente vascular cerebral e doença arterial periférica, respectivamente, estratificadas por sexo — homens (vermelho) e mulheres (azul). Os pontos representam os dados observados de 1990 a 2021, enquanto as linhas correspondem às projeções para 2022–2040, com base em modelos bayesianos idade-período-coorte. As linhas contínuas indicam as estimativas medianas; as linhas tracejadas representam o intervalo interquartil (25º ao 75º percentil); e as linhas pontilhadas refletem o intervalo de incerteza de 95% (2,5º ao 97,5º percentil).

Entre 1990 e 2021, a mortalidade padronizada por idade por DIC caiu tanto em homens quanto em mulheres, embora as taxas tenham se mantido consistentemente mais altas entre os homens (Tabela S1). Entre 2021 e 2040, projeta-se que a taxa padronizada por idade por DIC diminua 25,33% em homens, mas aumente 4,13% em mulheres (Tabela S2, Figura 3 e Figura 4). Conforme mostrado na Figura 4, a diferença na mortalidade por DIC entre homens e mulheres deverá se estreitar ao longo do tempo.

De forma semelhante, a mortalidade padronizada por idade por AVC também apresentou declínio substancial entre 1990 e 2021 (Tabela S3, Figura S2). Espera-se que essa tendência de queda persista até 2040, com uma redução global de 17,36% no Brasil (Tabela 1, Figura 1). Entre os grupos etários, a maior queda é projetada para indivíduos de 50-54 anos (Tabela 1, Figura S4, Figura 2).

De 1990 a 2021, as taxas de mortalidade por AVC diminuíram em ambos os sexos (Tabela S3). Entre 2021 e 2040, novas reduções são projetadas; no entanto, a queda será significativamente maior entre os homens (30,02%) em comparação às mulheres (4,51%) (Tabela S4, Figura 3 e Figura 4). Como ilustrado na Figura 4, a diferença entre os sexos na mortalidade por AVC também deverá se reduzir ao longo do tempo.

Com relação à DAP, a taxa de mortalidade padronizada por idade apresentou declínio inicial entre 1990 e 2015, seguido por um aumento acentuado até 2021 (Tabela S5, Figura S2). Entre 2021 e 2040, projeta-se que a mortalidade por DAP no Brasil aumente 10,99% (Tabela 1, Figura 1). Notadamente, as tendências projetadas variam substancialmente por faixa etária. Enquanto se espera uma redução de 7,69% no grupo de 40–44 anos, a mortalidade deverá aumentar 34,35% entre os indivíduos de 75 a 79 anos (Tabela 1, Figura S5, Figura 2).

A tendência de queda inicial seguida por aumento acentuado na mortalidade por DAP foi observada tanto em homens quanto em mulheres. Ao longo de todo o período observado, os homens apresentaram taxas consistentemente mais elevadas de mortalidade por DAP (Tabela S5). Entre 2021 e 2040, projeta-se que a mortalidade padronizada por idade por DAP aumente 14,61% em homens e 21,99% em mulheres (Tabela S6, Figura 3 e Figura 4).

A Figura Central fornece um resumo gráfico dos principais achados do estudo e da abordagem metodológica empregada.

## Discussão

Examinamos a carga observada de mortalidade por DIC, AVC e DAP entre adultos brasileiros de 1990 a 2021 e ajustamos modelos para projetar estimativas futuras até 2040. Nossos achados indicam que as taxas de mortalidade padronizadas por idade para DIC e AVC vêm diminuindo de forma contínua nas últimas 3 décadas, com projeções que apontam para a manutenção dessa tendência. Em contraste, espera-se que a mortalidade por DAP aumente entre 2021 e 2040, com um crescimento desproporcionalmente maior entre as mulheres.

A redução observada na mortalidade por DIC e AVC desde 1990 está em consonância com achados prévios relatados por Bastos et al., que observaram tendências semelhantes, mas destacaram que as melhorias foram atenuadas em regiões socioeconomicamente vulneráveis, especialmente nas regiões Norte e Nordeste do Brasil.^20^ A Sociedade Brasileira de Cardiologia (SBC) também relatou reduções contínuas na mortalidade por DIC e AVC, apesar do impacto adverso da COVID-19 nos desfechos cardiovasculares.^3,21^ Projeções comparáveis foram publicadas para países como Estados Unidos, Reino Unido e Japão.^9,10,22^ Em conjunto com nossos achados, esse corpo de evidências é encorajador, pois sugere que a mortalidade por DIC e AVC pode continuar a declinar, mesmo diante do expressivo envelhecimento populacional.

No entanto, nosso estudo também revelou que a queda projetada na mortalidade por AVC será consideravelmente menor entre os idosos. Um estudo anterior com dados dos EUA relatou reduções mínimas na mortalidade por AVC entre indivíduos com 65-74, 75-84 e 85 anos ou mais, de ambos os sexos.^9^ Esses achados ressaltam a necessidade de estratégias específicas de prevenção e cuidados com o AVC nas populações idosas. É importante destacar que DIC e AVC compartilham diversos fatores de risco modificáveis. Uma meta-análise recente envolvendo 50.000 pacientes mostrou que aproximadamente um terço dos pacientes com AVC agudo também apresenta DIC e possui risco aumentado de infarto do miocárdio dentro de 1 ano após o AVC.^23^

No Brasil, o cuidado agudo do AVC é dificultado por populações rurais extensas e pela distribuição desigual dos recursos de saúde. Embora unidades dedicadas ao atendimento do AVC e programas de fibrinólise tenham sido implementados em diversos centros metropolitanos, o acesso permanece limitado em regiões rurais e desassistidas.^24^ Embora as políticas públicas de saúde existentes tenham contribuído para a melhora da mortalidade por DIC e AVC, esforços adicionais são necessários para consolidar esses avanços — especialmente diante do rápido envelhecimento da população brasileira.

Nosso estudo também destaca 2 aspectos particularmente preocupantes: (i) a crescente carga de mortalidade por DAP no Brasil e (ii) diferenças significativas entre os sexos nas taxas projetadas de mortalidade por DIC, AVC e DAP. Segundo o GBD, a carga global de DAP aumentou 72% entre 1990 e 2019.^25^ Projeções do *Institute for Health Metrics and Evaluation* — responsável pelo GBD — indicam que os países de baixa e média renda deverão arcar com uma parcela desproporcionalmente maior dessa carga.^2^ A DAP avançada está associada a elevadas taxas de amputação de membros inferiores, baixa qualidade de vida, morbidade substancial e mortalidade elevada em 5 a 10 anos.^27,28^ Esses desfechos ressaltam a urgência de implementação de políticas públicas voltadas para a detecção precoce e o manejo adequado da DAP.

A segunda questão-chave identificada refere-se à acentuada disparidade nos desfechos cardiovasculares entre os sexos. Um estudo realizado na Espanha observou que, em comparação aos homens, as mulheres apresentaram maiores atrasos no diagnóstico e tratamento do infarto agudo do miocárdio.^28^ No contexto da DAP, os desfechos da cirurgia vascular também são piores para as mulheres, que têm maior probabilidade de se apresentar com isquemia aguda de membros.^26,29^ Segundo o relatório de 2023 do Ministério da Saúde do Brasil e da SBC, as taxas de obesidade são mais elevadas entre mulheres do que entre homens, e as mulheres têm 44% mais probabilidade de apresentar dislipidemia.^3^ A maior prevalência e mortalidade por DAP entre mulheres pode também refletir a presença de aterosclerose não diagnosticada ou não tratada, condição sabidamente mais prevalente entre mulheres. Em conjunto, esses achados reforçam a necessidade urgente de iniciativas de saúde direcionadas à redução das disparidades de sexo nos desfechos cardiovasculares.

Em 2011, o Ministério da Saúde do Brasil lançou um plano estratégico de 10 anos para redução das taxas de doenças crônicas não transmissíveis. Desde então, o país obteve conquistas importantes, incluindo uma redução de 30% na prevalência de tabagismo e um aumento de 10% na prática de atividade física entre adultos.^30^ Esses avanços são expressivos, considerando que aproximadamente 70% da carga total de DCVs é atribuível a fatores de risco modificáveis.^2^ No entanto, como observado em muitos outros países, o Brasil continua enfrentando aumento nas taxas de obesidade em adultos, bem como maior prevalência de hipertensão, diabetes e dislipidemia.^30^

Em resposta, o governo brasileiro — em parceria com a SBC e outras sociedades médicas — apresentou, em 2023, um novo plano nacional de enfrentamento das DCVs, atualmente em análise pelo Senado.^31^ Essa proposta legislativa busca enfrentar os desafios atuais por meio da implementação de intervenções direcionadas e da criação de um fundo federal dedicado ao combate das DCV. A alocação proposta para esse fundo inclui: 30% para pesquisa relacionada às DCV; 15% para campanhas públicas de conscientização sobre fatores de risco cardiovasculares; 30% para capacitação de profissionais de saúde, com ênfase na atenção primária; e 15% para programas voltados à redução do tabagismo, do consumo de álcool e de outros fatores de risco modificáveis. Além disso, 10% do fundo seriam administrados pelo Conselho Federal de Medicina para apoiar a formação especializada em cuidados cardiovasculares. Tais iniciativas podem contribuir para reverter as tendências recentes de subfinanciamento do sistema público de saúde no Brasil.^32^

Melhorar a adesão ao tratamento medicamentoso e ampliar o acesso aos cuidados — especialmente por meio da formação e distribuição geográfica de profissionais de saúde capacitados em cardiologia preventiva — também são estratégias fundamentais para enfrentar de forma eficaz a carga nacional de DCV.^5,33,34^

Além dos fatores de risco cardiovasculares tradicionais, é essencial reconhecer o papel das exposições ambientais, como a poluição do ar por partículas finas, que foi associada causalmente às DCV. De forma encorajadora, dados recentes mostram que a mortalidade atribuível à poluição do ar no Brasil diminuiu entre 2010 e 2019.^35^ Contudo, o mesmo estudo relatou que os níveis de poluição em determinadas regiões permaneceram múltiplas vezes acima dos limites recomendados pela OMS.^35^ Assim, os futuros esforços em políticas públicas devem não apenas manter o foco nos fatores de risco convencionais para DCV, mas também se expandir para contemplar os determinantes ambientais da saúde cardiovascular.

Por fim, conforme amplamente discutido na comunidade cardiológica, o desenvolvimento de diretrizes de cardiologia preventiva especificamente voltadas às mulheres constitui um passo crucial para reduzir as disparidades de sexo identificadas em nosso estudo.^36-39^

### Pontos fortes e limitações

Este estudo apresenta várias limitações que devem ser reconhecidas. Tanto as taxas de eventos quanto as estimativas populacionais de meio de ano foram obtidas a partir do conjunto de dados do GBD 2021. Embora esses dados tenham como fonte primeira o SIM, o GBD aplica seus próprios procedimentos de modelagem. Como resultado, nossos achados podem divergir de estudos que extraem diretamente os dados da fonte original.^40,41^ Ainda assim, por harmonizar os dados entre países, o GBD permite que nossos resultados sejam mais facilmente comparáveis a estudos internacionais.

Embora o SIM realize rotineiramente verificações de qualidade para garantir a precisão dos dados, os atestados de óbito permanecem como principal fonte de informação sobre mortalidade. A acurácia desses registros pode variar, especialmente em regiões rurais com recursos limitados. No entanto, a metodologia do GBD é especificamente desenvolvida para lidar com questões de completude e classificação incorreta dos dados.

Nosso estudo não considera fatores de risco em nível individual ou populacional. Tendências futuras na mortalidade por DIC, AVC e DAP podem ser significativamente influenciadas por alterações nesses fatores. Embora estratégias mais agressivas de mitigação possam reduzir as taxas de eventos no futuro, as tendências atuais — como o aumento da prevalência de obesidade, diabetes e tabagismo — podem potencialmente resultar em mortalidade ainda mais elevada do que a projetada.

Utilizamos modelos bayesianos IPC para projetar estimativas futuras de mortalidade. Como toda abordagem de modelagem, esses modelos requerem certas premissas. Embora não se possa descartar a possibilidade de especificação inadequada, apresentamos II 95% para contemplar tais limitações. Ademais, os desfechos em saúde pública são influenciados por uma ampla gama de fatores — incluindo mudanças políticas, culturais, migratórias e ambientais — muitos dos quais não podem ser totalmente capturados pelos modelos matemáticos utilizados neste estudo.

## Conclusão

Com base nos dados observados de 1990 a 2021, projeta-se que a mortalidade padronizada por idade por DIC e AVC no Brasil continue em declínio até 2040. Em contraste, espera-se que a mortalidade padronizada por idade atribuível à DAP aumente substancialmente ao longo do mesmo período. Enquanto as reduções projetadas na mortalidade por DIC e AVC serão mais acentuadas entre os homens, o aumento na mortalidade por DAP deverá ser consideravelmente maior entre as mulheres. Esses achados ressaltam a necessidade urgente de intervenções sustentadas e direcionadas em saúde pública, com o objetivo de reduzir disparidades cardiovasculares, ampliar a conscientização e melhorar o acesso equitativo a cuidados embasados em evidências em todo o Brasil.

## *Material suplementar

Para informação adicional, por favor, clique aqui.https://abccardiol.org/supplementary-material/2025/12212/2025-0189_final_supplemental_section.pdf
