# Supplementary material for: Projeções de Mortalidade por Doença Isquêmica do Coração, Acidente Vascular Cerebral e Doença Arterial Periférica no Brasil até 2040: Uma Abordagem de Modelagem Bayesiana
Source: Arq Bras Cardiol. 2025 Dec 18;122(11):e20250189. [Article in Portuguese] doi: 10.36660/abc.20250189 (PMC12978281; doi:10.36660/abc.20250189)
Supplement: Supplementary file 1 [file 0066-782x-abc-122-11-e20250189-suppl01.pdf]

1. Data sources

A thorough description of the definition and methodologies used by GBD to estimate each component's burden has been previously described.[1] In brief, for IHD burden, GDB considers acute myocardial infarction, chronic stable angina, chronic IHD, and heart failure due to IHD.[1] GBD adopted the Fourth Universal Definition of Myocardial Infarction but also included out-of-hospital sudden cardiac death as myocardial infarction.[1] The Rose Angina Questionnaire was used to define stable angina and stroke was defined using the World Health Organization definition.[1] Additionally, PAD was defined by an ankle-brachial index <0.9. [1]

In GBD, mortality data sources included vital registration data coded to the International Classification of Disease (ICD) system or household mortality surveys known as verbal autopsy.[1] For Brazil, the main source of cardiovascular mortality data was the Sistema the Informações sobre Mortalidade, the national Brazilian vital registration system maintained by its Ministry of Health.[2] Several statistical methods were employed by GBD to harmonize mortality data sources, these methods encompassed the reclassification of nonspecific or unspecified codes, noise reduction algorithms, and the utilization of Bayesian geospatial regression software called CODem (Cause of Death Ensemble Model), developed by the Institute for Health Metrics and Evaluation in Seattle, Washington.[1] CODem leveraged location-specific covariates to generate smoothed time trends for 204 countries and territories, employing strength borrowing across age, space, and time dimensions.[1]

To adopt a consistent source for our data, we opted to obtain our mid-year population estimates from 1990-2040 from the GBD database. This dataset draws from 1,250 censuses and 747 population registry location-year.[3] For Brazil, the main source of primary data is the Brazilian census performed by the Brazilian Institute of Geography and Statistics. Additional information regarding how GBD obtains and processes population estimates can be found in the original paper.[4]

2. Bayesian Age Period Cohort Models:

Age-Period-Cohort models are recommended by demographers as the correct method to evaluate temporal changes in data & project future rates. They suffer from an identifiability problem as there is an exact linear relationship between age, period, and cohort as one can be directly calculated from the other two. Due to this linear dependence, it is impossible to identify separate contributions of the age, period and cohort effects. The observed event rate for the data can be presented as:

$$log(\lambda_{ij}) = \alpha(age - i) + \beta(period - j) + \gamma(cohort) + \mu\,(intercept\ term)$$

While the identifiability problem can make it challenging to identify individual contributions of age, period and cohort, accurately projecting future rates is more easily possible. However, in this study, our primary aim was to be able to reliably project future event rates, which can be reliably identified using these models. The Bayesian age-period-cohort models also model non-linear changes in the observed data. However, unlike, frequentist approaches, where spline terms need to be fitted with predetermined knots or polynomial terms need to be chosen a-priori, this model does not need such inputs from the user. Hence, it chooses an equation that is best suited to the data.

Model priors: We used smoothing priors for the age, period and cohort effects. The standard choice is to use the second-order random walk (RW2) which assumes independent mean-zero normal distributions (with unknown variance) on the second differences of all time effects. The RW2 smoothing prior can be presented as: $\Delta^2\theta_i = \theta_i - 2\theta_{i-1} + \theta_{i-2} \sim N(0, \sigma^2)$ where θ represents the parameter for the i-th level. The benefits of using the RW2 prior are: 1. It helps smooth the estimates effects across adjacent periods and reduces the noise and improves interpretability. 2. It breaks the perfect collinearity between the three effects and therefore all three effects can be simultaneously introduced into the model.

Model convergence and diagnostic accuracy: We used the Integrated Network Laplace Approximation (INLA) method to approximate the posterior marginal distributions directly from the model without Markov Chain Monte Carlo (MCMC) sampling method. The INLA approach is faster, does not have any convergence concerns, and provides very similar results to the traditional MCMC approach. The model's predictive accuracy was checked by calculating the Brier score, root mean square error and mean absolute error from the observed and model predicted values between 1990 - 2021. Additionally, we graphed and compared the observed and predicted estimates between 1990 -2021 for concordance.

We refer readers to the following manuscripts for further information regarding the BAPC package and fitting BAPC models using INLA in R:
1. https://rdrr.io/rforge/BAPC/ - this webpage provides information regarding the functions present in the BAPC package.
2. Riebler A, Held L. Projecting the future burden of cancer: Bayesian age-period-cohort analysis with integrated nested Laplace approximations. Biom J. 2017 May;59(3):531-549. doi: 10.1002/bimj.201500263. Epub 2017 Jan 31. PMID: 28139001. – This is their manuscript wherein they present the details of their modeling process.
3. Supplemental material to the above paper provides R code used for the examples presented in their manuscript.
4. https://cran.r-project.org/web/packages/scoringRules/index.html - We used the scoringRules R package to calculate the model metrics

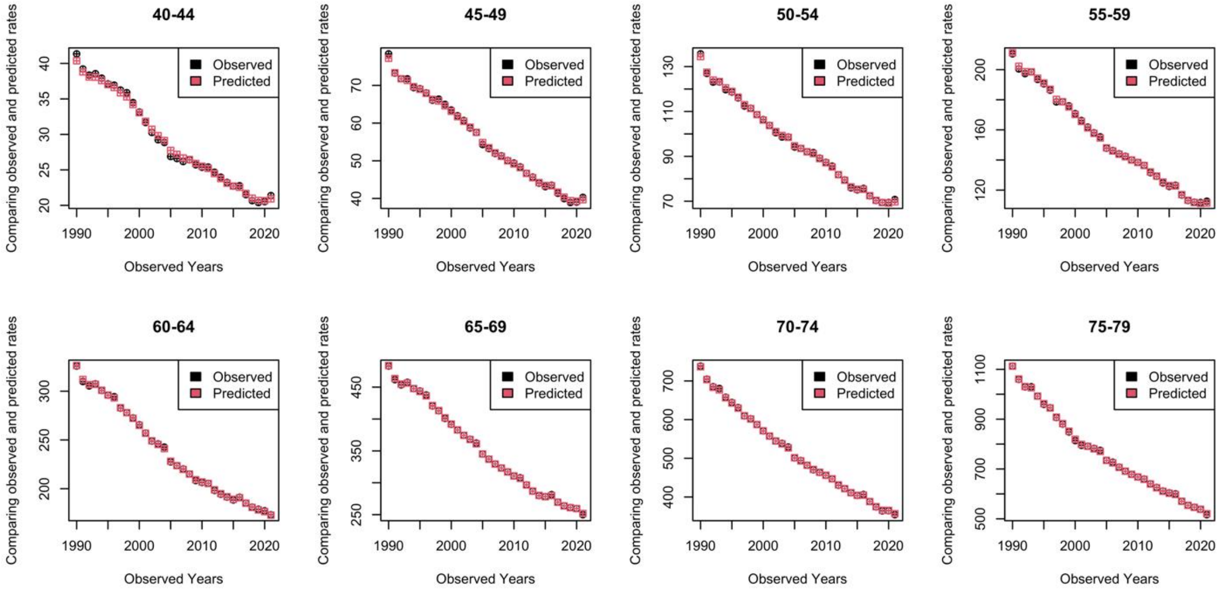

Figure 2.1: This plot presents the observed and predicted mortality rates for ischemic heart disease for each studied age bracket. The predicted rates were obtained from the fitted Bayesian age-period-cohort model. As depicted, the difference between observed and predicted rates is small.

Table 2.1: Model scoring table- ischemic heart disease:

Age Bracket	MAE	RMSE	Brier Score
40-44 years	0.0000032249	0.0000039092	0.0000000000
45-49 years	0.0000026889	0.0000034727	0.0000000000
50-54 years	0.0000034200	0.0000045891	0.0000000000
55-59 years	0.0000052705	0.0000067814	0.0000000000
60-64 years	0.0000055833	0.0000071512	0.0000000001
65-69 years	0.0000056897	0.0000077869	0.0000000001
70-74 years	0.0000093751	0.0000125207	0.0000000002
75-79 years	0.0000154967	0.0000210792	0.0000000004

This table presents the scoring tests to compare the observed rates (1990-2021) and predicted rates obtained from the Bayesian age-period-cohort model for each age bracket. A smaller value signifies better concordance between the observed and predicted values.
MAE – Mean absolute error, RMSE – root mean square error.

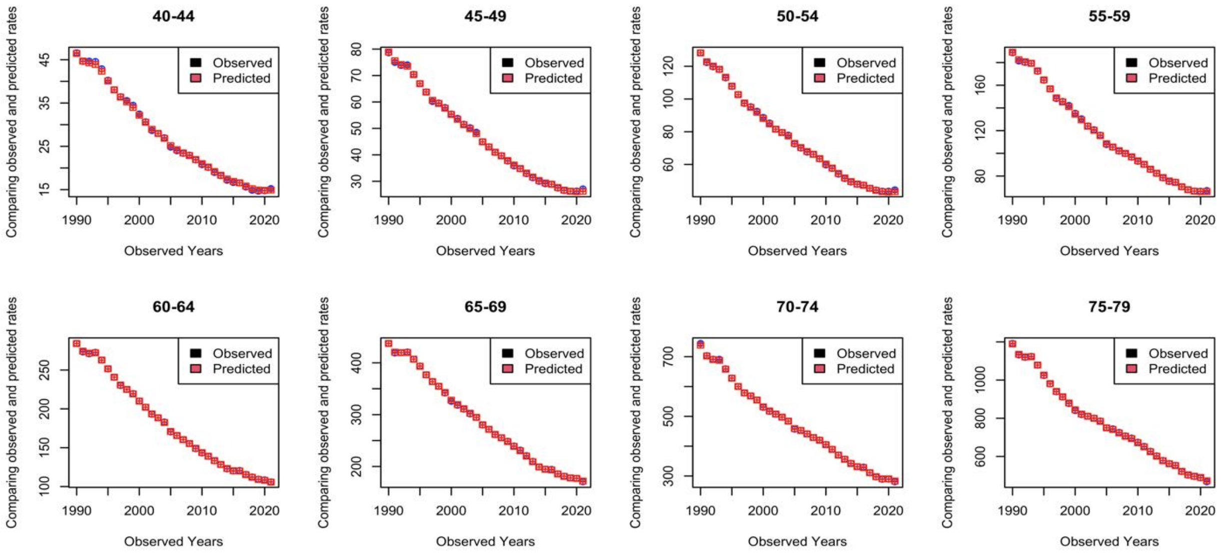

Figure 2.2: This plot presents the observed and predicted mortality rates for stroke for each studied age bracket. The predicted rates were obtained from the fitted Bayesian age-period-cohort model. As depicted, the difference between observed and predicted rates is small.

Table 2.2: Model scoring table – Stoke:

Age Bracket	MAE	RMSE	Brier Score
40-44 years	0.0000022313	0.0000027253	0.0000000000
45-49 years	0.0000024738	0.0000031108	0.0000000000
50-54 years	0.0000032488	0.0000039414	0.0000000000
55-59 years	0.0000041731	0.0000050482	0.0000000000
60-64 years	0.0000037672	0.0000045192	0.0000000000
65-69 years	0.0000062216	0.0000082111	0.0000000001
70-74 years	0.0000109408	0.0000158522	0.0000000003
75-79 years	0.0000216234	0.0000240567	0.0000000006

This table presents the scoring tests to compare the observed rates (1990-2021) and predicted rates obtained from the Bayesian age-period-cohort model for each age bracket. A smaller value signifies better concordance between the observed and predicted values.
MAE – Mean absolute error, RMSE – root mean square error.

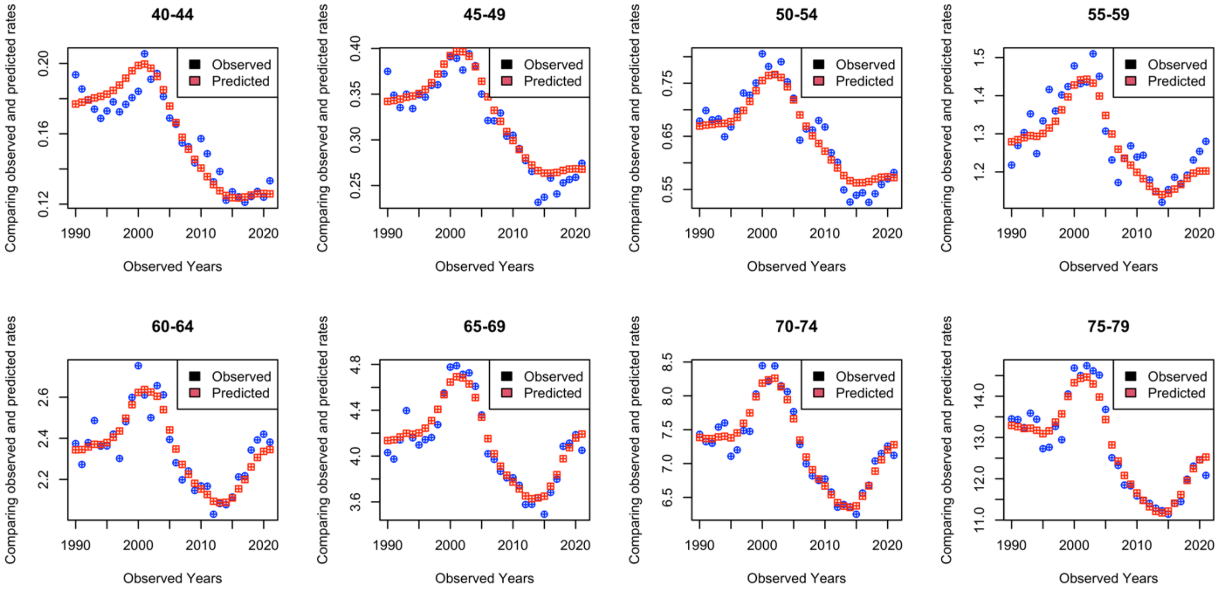

Figure 2.3: This plot presents the observed and predicted mortality rates for peripheral artery disease for each studied age bracket. The predicted rates were obtained from the fitted Bayesian age-period-cohort model. As depicted, the difference between observed and predicted rates is small.

Table 2.3: Model scoring table – Peripheral artery disease:

Age Bracket	MAE	RMSE	Brier score
40-44 years	0.0000000673	0.0000000864	0.0000000000
45-49 years	0.0000001083	0.0000001407	0.0000000000
50-54 years	0.0000002030	0.0000002471	0.0000000000
55-59 years	0.0000003805	0.0000004630	0.0000000000
60-64 years	0.0000005031	0.0000006289	0.0000000000
65-69 years	0.0000007861	0.0000009426	0.0000000000
70-74 years	0.0000010317	0.0000012933	0.0000000000
75-79 years	0.0000019021	0.0000025154	0.0000000000

This table presents the scoring tests to compare the observed rates (1990-2021) and predicted rates obtained from the Bayesian age-period-cohort model for each age bracket. A smaller value signifies better concordance between the observed and predicted values.
MAE – Mean absolute error, RMSE – root mean square error.

3. Supplemental Tables:

Table S1: Age-standardized IHD mortality rates for Brazil

Year	Overall			Males			Females		
	Crude rate	Age-standardized rate (mean, 95% CI)		Crude rate	Age-standardized rate (mean, 95% CI)		Crude rate	Age-standardized rate (mean, 95% CI)	
1990	234.86	234.86	233.21, 236.53	303.40	303.40	300.69, 306.13	170.82	168.31	166.37, 170.26
1995	208.07	208.92	207.47, 210.37	265.83	267.79	265.42, 270.18	155.01	152.86	151.16, 154.57
2000	184.06	184.83	183.58, 186.09	238.71	241.27	239.19, 243.36	134.44	132.13	130.68, 133.59
2005	161.77	161.59	160.51, 162.67	211.20	212.92	211.12, 214.74	117.27	114.27	113.04, 115.51
2010	150.59	147.65	146.71, 148.61	199.65	197.45	195.85, 199.05	106.56	102.10	101.03, 103.18
2015	139.7	131.72	130.9, 132.55	186.25	177.02	175.63, 178.42	98.04	90.28	89.35, 91.21
2021	130.8	118.61	117.9, 119.32	173.66	159.25	158.04, 160.46	92.56	81.58	80.78, 82.38

This table reports the age-standardized (per 100,000 individuals) with 95% confidence interval (CI) and crude rates (per 100,000 individuals) for ischemic heart disease (IHD) mortality for Brazil between 1990 and 2021.

Table S2: Projected age standardized IHD mortality rates for Brazil

Year	Overall	Males	Females
2022	118.20 (114.73, 121.67)	157.48 (153.65, 161.3)	82.82 (79.98, 85.67)
2025	115.33 (101.58, 129.07)	150.27 (138.63, 161.9)	83.27 (72.09, 94.45)
2030	110.76 (72.36, 149.16)	139.04 (109.58, 168.5)	84.10 (51.71, 116.48)
2035	106.08 (37.81, 174.34)	128.33 (78.72, 177.95)	84.63 (24.42, 144.83)
2040	101.82 (0.36, 203.27)	118.91 (48.19, 189.64)	84.95 (0, 178.3)
Percent Change (2021-2040)	-14.16%	-25.33%	+4.13%
Estimated Annual Percent Change (2021-2040)	-0.83 (-0.84, -0.83) %	-1.56 (-1.57, -1.55) %	0.14 (0.13, 0.16) %

This table reports the projected median and 95% uncertainty interval for age-standardized ischemic heart disease (IHD) rates (per 100,000) in Brazil between 2022 and 2040.

Table S3: Age-standardized stroke mortality rates for Brazil

	Overall			Males			Females		
Year	Crude rate	Age-standardized		Crude rate	Age-standardized rate		Crude rate	Age-standardized rate	
1990	227.31	227.31	225.68, 228.95	263.53	263.53	261.00, 266.07	193.49	191.11	189.04, 193.19
1995	196.12	196.77	195.36, 198.18	226.07	227.62	225.44, 229.82	168.59	166.64	164.86, 168.43
2000	162.79	162.99	161.82, 164.17	189.01	190.59	188.75, 192.45	138.98	136.67	135.20, 138.16
2005	138	137.21	136.22, 138.2	159.57	160.51	158.95, 162.08	118.56	115.28	114.04, 116.52
2010	121.11	118.38	117.53, 119.23	142.54	140.86	139.51, 142.21	101.86	97.41	96.37, 98.47
2015	103.32	97.06	96.36, 97.77	121.50	115.00	113.89, 116.13	87.05	80.24	79.37, 81.12
2021	93.88	84.58	83.99, 85.18	110.40	100.15	99.20, 101.11	79.15	70.14	69.39, 70.89

This table reports the age-standardized (per 100,000 individuals) with 95% confidence interval (CI) and crude rates (per 100,000 individuals) for stroke mortality for Brazil between 1990 and 2021.

Table S4: Projected age-standardized stroke mortality rates for Brazil

Year	Overall	Males	Females
2022	84.67 (81.76, 87.58)	99.41 (96.08, 102.73)	71.05 (68.52, 73.59)
2025	82.30 (70.71, 93.90)	94.13 (82.52, 105.74)	70.64 (61.16, 80.11)
2030	78.38 (46.18, 110.59)	85.73 (55.68, 115.77)	69.9 (43.28, 96.52)
2035	74.06 (17.56, 130.56)	77.47 (27.44, 127.49)	68.58 (20.47, 116.7)
2040	69.90 (0, 152.48)	70.09 (0.26, 139.92)	66.98 (0, 139.45)
Percent Change (2021-2040)	**-17.36%**	**-30.02%**	**-4.51%**
**Estimated Annual Percent Change (2021-2040)**	-1.07 (-1.10, -1.04) %	-1.94 (-1.96, -1.91) %	-0.33 (-0.37, -0.29) %

This table reports the projected median and 95% uncertainty intervals for age-standardized stroke rates (per 100,000) in Brazil between 2022 and 2040.

Table S5: Age-standardized PAD mortality rates for Brazil

	Overall			Males			Females		
Year	Crude rate	Age-standardized rate		Crude rate	Age-standardized rate		Crude rate	Age-standardized rate	
1990	1.93	1.93	1.78, 2.09	2.43	2.43	2.20, 2.69	1.45	1.42	1.25, 1.61
1995	1.88	1.89	1.76, 2.03	2.35	2.37	2.15, 2.60	1.45	1.42	1.26, 1.60
2000	2.20	2.19	2.06, 2.33	2.70	2.72	2.51, 2.95	1.74	1.70	1.54, 1.87
2005	2.01	2.00	1.88, 2.12	2.51	2.53	2.33, 2.73	1.58	1.52	1.38, 1.66
2010	1.80	1.76	1.66, 1.86	2.23	2.21	2.04, 2.38	1.41	1.34	1.22, 1.47
2015	1.75	1.63	1.54, 1.72	2.14	2.01	1.87, 2.17	1.39	1.27	1.16, 1.38
2021	2.06	1.82	1.73, 1.91	2.54	2.26	2.12, 2.41	1.64	1.41	1.31, 1.52

This table reports the age-standardized (per 100,000 individuals) with 95% confidence interval (CI) and crude rates (per 100,000 individuals) for peripheral artery disease (PAD) mortality for Brazil between 1990 and 2021.

Table S6: Projected age-standardized PAD mortality rates for Brazil

Year	Overall	Males	Females
2022	1.87 (1.73, 2.01)	2.35 (2.15, 2.54)	1.46 (1.33, 1.6)
2025	1.93 (1.52, 2.34)	2.44 (1.91, 2.97)	1.52 (1.17, 1.88)
2030	2.01 (0.91, 3.10)	2.56 (1.18, 3.94)	1.62 (0.68, 2.55)
2035	2.04 (0.04, 4.03)	2.61 (0.13, 5.08)	1.68 (0, 3.39)
2040	2.02 (0, 5.03)	2.59 (0, 6.31)	1.72 (0, 4.34)
Percent Change (2021-2040)	+10.99%	+14.6%	+21.99%
Estimated Annual Percent Change (2021-2040)	0.45 (0.30, 0.59) %	0.55 (0.38, 0.71) %	0.91 (0.78, 1.02) %

This table reports the projected median and 95% uncertainty intervals for age-standardized peripheral artery disease (PAD) rates (per 100,000) in Brazil between 2022 and 2040.

4. Supplemental Figures

Figure S1. The mid-year population (40-79 years) for Brazil (1990-2040).

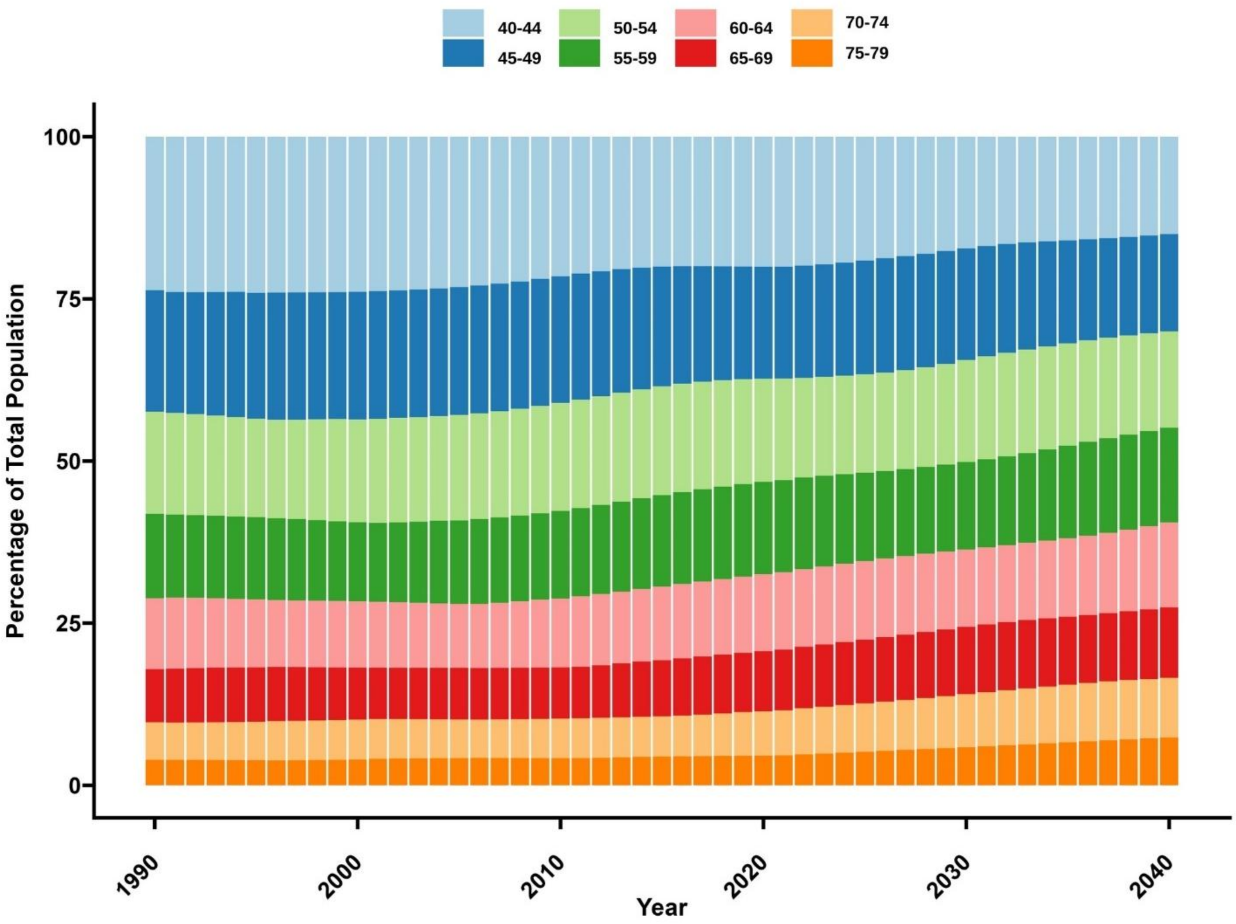

Between 1990 and 2040 the Brazilian population will age; the proportion of Brazilians in the 40-44 age group will decline from 23.66% to 15.00% while the proportion of the individuals in the 75-79 group will increase from 3.93% to 7.38%.

Figure S2. Crude and age standardized mortality rates for IHD, stroke and PAD in Brazil

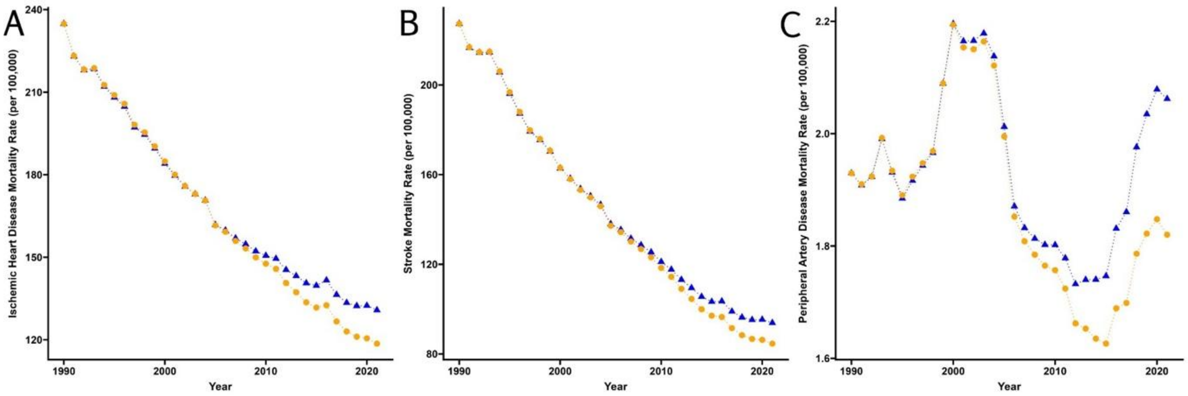

Crude (blue triangles) and age-standardized (yellow circles) mortality rates (per 100,000) of ischemic heart disease (IHD) (A), stroke (B), and peripheral artery disease (PAD) (C) among 40-79-year-old residents (1990 - 2021) in Brazil. The numerical values for the age standardized rates are provides in tables S1 (IHD), S3(Stroke), and S5(PAD).

Figure S3. IHD mortality rates for Brazil in each age bracket

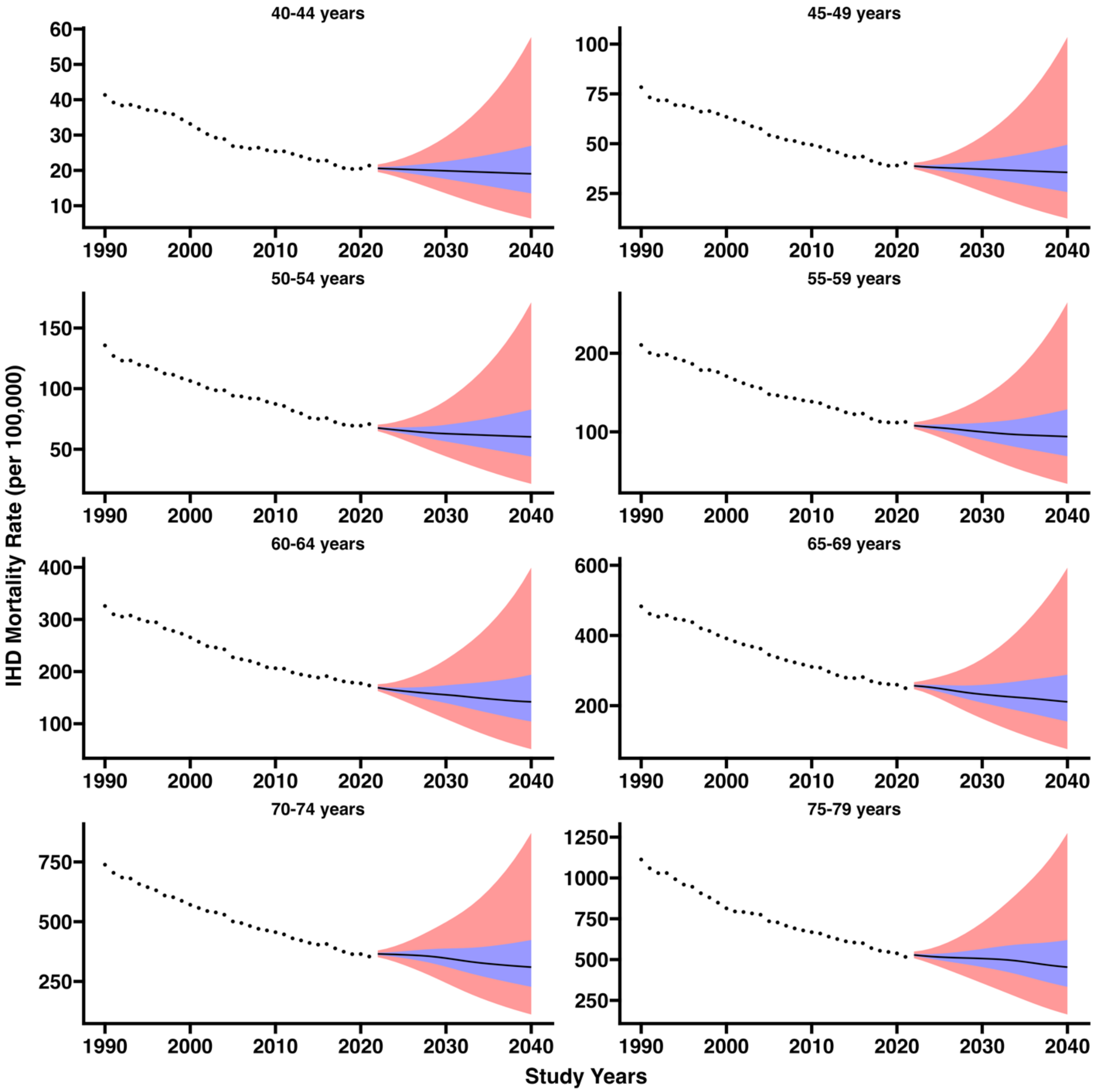

This figure shows the IHD mortality rates (per 100,000 individuals) for Brazil between 1990-2040 for each age bracket. The observed rates (1990-2021) are depicted as points while the projected estimates (2022 – 2040) are presented as a continuous line (median value). The fan plot depicts the projected uncertainty interval; the blue color represents the values between the 25th and 75th percentile. The combined red and blue colors represent all values that fall within the 95% uncertainty interval for the fitted model. *IHD – ischemic heart disease;* The numerical estimates for the median, 95% uncertainty interval are presented in table 1.

Figure S4. Stroke mortality rates for Brazil in each age bracket

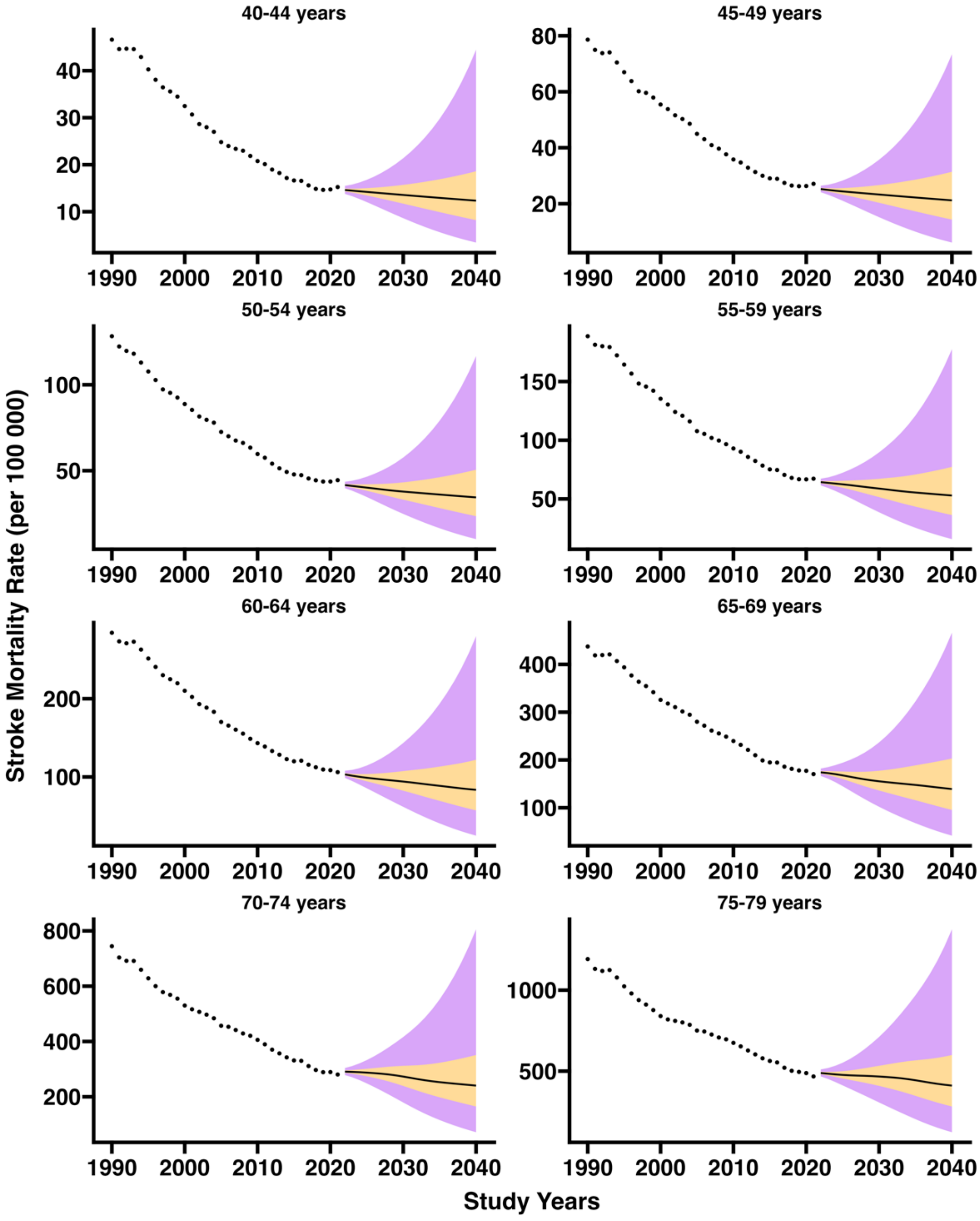

This figure shows the stroke mortality rates (per 100,000 individuals) for Brazil between 1990-2040 for each age bracket (the unit of age is years). The observed rates (1990-2021) are depicted as points while the projected median estimates (2022 – 2040) are presented as a continuous line (median value). The fan plot depicts the projected uncertainty interval; the orange color represents the values between the 25th and 75th percentile. The combined orange and purple colors represent all values that fall within the 95% uncertainty interval for the fitted model. The numerical estimates for the median, 95% uncertainty interval are presented in table 1.

Figure S5. PAD mortality rates for Brazil in each age bracket.

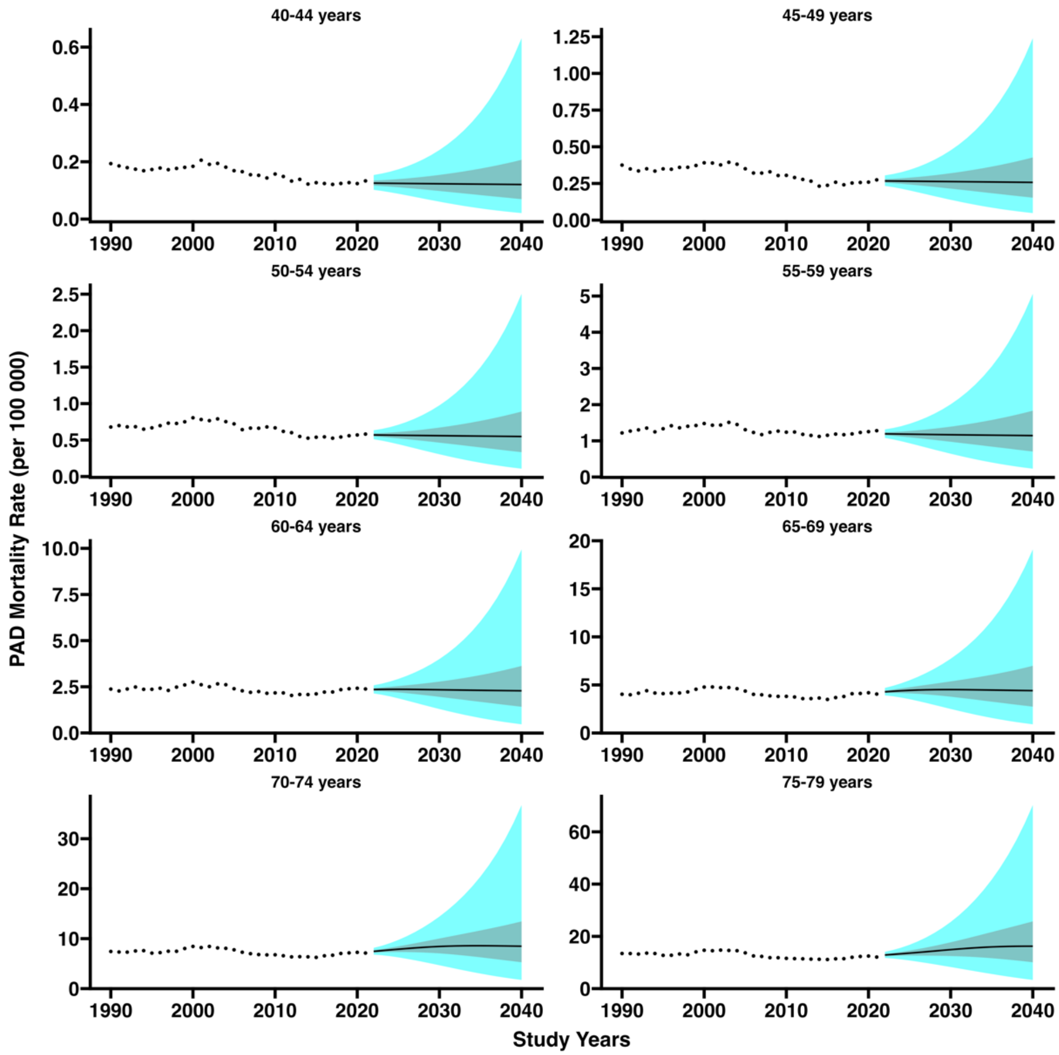

This figure shows the PAD mortality rates (per 100,000 individuals) for Brazil between 1990-2040 for each age bracket (the unit of age is years). The observed rates (1990-2021) are depicted as points while the projected median estimates (2022 – 2040) are presented as a continuous line (median value). The fan plot depicts the projected uncertainty interval; the darker cyan color represents the values between the 25th and 75th percentile. The combined light and dark cyan colors represent all values that fall within the 95% uncertainty interval for the fitted model. *PAD – peripheral artery disease; numerical estimates of the median, 95% uncertainty interval are present in table 1.*

References

1. Roth GA, Mensah GA, Johnson CO, Addolorato G, Ammirati E, Baddour LM, et al. Global Burden of Cardiovascular Diseases and Risk Factors, 1990–2019. J Am Coll Cardiol. 2020 Dec 22;76(25):2982–3021.

2. Global Burden of Disease Study 2019 (GBD 2019) Data Input Sources Tool | GHDx [Internet]. [cited 2024 Mar 25]. Available from: https://ghdx.healthdata.org/gbd-2019/data-input-sources?components=4&causes=491&locations=135

3. Institute for Health Metrics and Evaluation (IHME). Global Fertility, Mortality, Migration, and Population Forecasts 2017-2100 [Internet]. [object Object]; 2020 [cited 2024 Mar 25]. Available from: http://ghdx.healthdata.org/record/ihme-data/global-population-forecasts-2017-2100

4. Vollset SE, Goren E, Yuan CW, Cao J, Smith AE, Hsiao T, et al. Fertility, mortality, migration, and population scenarios for 195 countries and territories from 2017 to 2100: a forecasting analysis for the Global Burden of Disease Study. The Lancet. 2020 Oct 17;396(10258):1285–306.
