# Supplementary material for: Projeções de Mortalidade por Doença Isquêmica do Coração, Acidente Vascular Cerebral e Doença Arterial Periférica no Brasil até 2040: Uma Abordagem de Modelagem Bayesiana
Source: Arq Bras Cardiol. 2025 Dec 18;122(11):e20250189. [Article in Portuguese] doi: 10.36660/abc.20250189 (PMC12978281; doi:10.36660/abc.20250189)
Supplement: Translation

# Translation: Forecasting Ischemic Heart Disease, Stroke, and Peripheral Artery Disease Mortality in Brazil Through 2040: A Bayesian Modeling Approach

**DOI:** 10.36660/abc.20250189i

**Authors:** Pedro Rafael Vieira de Oliveira Salerno, Antoinette Cotton, Bruno R. Nascimento, Zhuo Chen, Gabriel Tensol Rodrigues Pereira, Alexandre A. Abizaid, Luis Augusto Palma Dallan, Pedro Rafael Salerno, Sadeer Al-Kindi, Fanny Petermann-Rocha, Salil V. Deo

**Affiliations:** 1 USA Department of Medicine, NYC Health + Hospitals/Elmhurst, Icahn School of Medicine at Mt. Sinai, Queens, NY – USA; 2 USA University Hospitals of Cleveland, Cleveland, Ohio – USA; 3 USA Case Western Reserve University, Cleveland – USA; 4 Brazil Universidade Federal de Minas Gerais, Belo Horizonte, MG – Brazil; 5 Brazil Hospital Israelita Albert Einstein, São Paulo, SP – Brazil; 6 Brazil Instituto do Coração do Hospital das Clínicas da Faculdade de Medicina da Universidade de São Paulo, São Paulo, SP – Brazil; 7 Brazil Universidade Católica de Pernambuco, Recife, PE – Brazil; 8 USA Center for Health and Nature Houston Methodist, Houston – USA; 9 Chile Universidad Diego Portales, Santiago – Chile; 10 USA Louis Stokes VA Medical Center, Cleveland, Ohio – USA; 11 England University of Glasgow, Glasgow – England

**Keywords:** Myocardial Ischemia, Stroke, Peripheral Arterial Disease, Mortality, Brazil

## Abstract

**Background:**

Atherosclerotic cardiovascular disease — particularly ischemic heart disease (IHD), stroke, and peripheral artery disease (PAD) — is the leading cause of cardiovascular mortality in Brazil.

**Objectives:**

This study aimed to project mortality trends for IHD, stroke, and PAD in Brazil through 2040.

**Methods:**

Annual death counts (1990-2021) for IHD, stroke, and PAD among individuals aged 40-79 years were obtained from the Global Burden of Disease (GBD) 2021 study. Mid-year population estimates for both observed (1990-2021) and projected (2022-2040) periods were used. Crude and age-standardized mortality rates were calculated. Bayesian age-period-cohort models were applied to project mortality rates from 2022 to 2040. Relative percentage changes and estimated annual percentage changes (EAPCs) were computed. Projections (per 100,000 population) are reported as medians with 95% uncertainty intervals (UIs), and EAPCs include bootstrap-derived confidence intervals (CIs).

**Results:**

Between 1990 and 2040, Brazil’s population aged 40-79 years is projected to grow by 237.82%. The age-standardized mortality rate for IHD is expected to decline by 14.16% [from 118.61 in 2021 to 101.82 in 2040 (95% UI, 0.36-203.27)] (EAPC: -0.83% [95% CI, -0.84 to -0.83]); and for stroke by 17.36% [from 84.58 to 69.90 (95% UI, 0-152.48)] (EAPC: -1.07% [95% CI, -1.10 to -1.04]). In contrast, PAD mortality is projected to increase by 10.99% [from 1.82 to 2.02 (95% UI, 0-5.03)] (EAPC: 0.45% [95% CI, 0.30-0.59]). Additionally, sex-specific age-standardized mortality rates showed considerable variation. For IHD, rates are projected to decline by 25.31% among men (EAPC: -1.56% [95% CI, -1.57 to -1.55]), while increasing by 4.12% among women (EAPC: 0.14% [95% CI, 0.13-0.16]). Stroke mortality is expected to decrease by 30.00% in men (EAPC: -1.94% [95% CI, -1.96 to -1.91]) and by 4.52% in women (EAPC: -0.33% [95% CI, -0.37 to -0.29]). In contrast, PAD mortality is projected to rise by 14.64% in men (EAPC: 0.55% [95% CI, 0.38 to 0.71]) and by 21.92% in women (EAPC: 0.91% [95% CI, 0.78-1.02]).

**Conclusion:**

While mortality rates for IHD and stroke are projected to decline, PAD mortality is expected to rise — particularly among women — highlighting the urgent need for sex-specific and disease-specific public health interventions.

## Introduction

Atherosclerotic cardiovascular disease (CVD) encompasses 3 major conditions: ischemic heart disease (IHD), stroke, and peripheral artery disease (PAD). In 2013, IHD and stroke together accounted for 247.9 deaths per 100,000 population worldwide.^1^ For many years, both have consistently ranked among the leading causes of cardiovascular mortality across most nations. In 2019, the Global Burden of Disease (GBD) study reported that 113 million adults globally were affected by PAD, with 42% of related deaths occurring in low- and middle-income countries, including Brazil.^2^ According to the Cardiovascular Statistics – Brazil 2023, CVD remains the leading cause of death in the country.^3^

Although the age-standardized mortality rate from CVD has declined over the past decades in Brazil, the prevalence of these conditions has increased by 26%.^4,5^ Rates of obesity and dyslipidemia remain high, and according to the 2024 report by the World Health Organization (WHO), Brazil is among the top-ranking countries in tobacco use. Moreover, mortality attributable to diabetes in the country is projected to rise by 144% over the next 2 decades.^4,6^

Recognizing the detrimental impact of cardiovascular conditions on national health, the Brazilian Congress enacted Law No. 8,080 in 1990, establishing the Brazilian Unified Health System (SUS), a free and universal public health care system. Currently, the SUS serves more than 70% of the Brazilian population.^7^ In addition, the Ministry of Health introduced the Family Health Program in the mid-1990s, followed by primary prevention strategies in 2002 and the implementation of a generic drug coverage policy for the treatment of hypertension, diabetes, and dyslipidemia.^8^ These initiatives have contributed to improvements in population health. However, as public policies evolve and therapeutic options advance, the attributable burden of CVD and its components may shift. Therefore, understanding the future trajectory of these conditions is critical to guiding national health strategies.

Some countries, such as the United States, Japan, and the United Kingdom, have already undertaken projections of future cardiovascular health.^9,10^ In the U.S. and Japan, IHD mortality is expected to decline, while stroke-related mortality is projected to remain relatively stable. However, such findings cannot be generalized to other contexts due to variations in population structure, fertility rates, aging patterns, and migration flows. Since Brazil is among the most populous countries globally, its contribution to global estimates is substantial.

Therefore, the present study aimed to project mortality rates for IHD, stroke, and PAD among Brazilian adults aged 40-79 years through 2040, based on observed data from 1990 to 2021.

## Methods

### Data source

#### IHD, stroke, and PAD mortality

The primary data source for this study was the GBD 2021 study.^11^ The GBD, a global consortium of over 12,000 collaborators, provides harmonized data on disability and mortality attributable to various health conditions and risk factors across 204 countries and territories.^12^ The 2021 update offers public access through the Global Health Data Exchange (GHDx) query tool (http://ghdx.healthdata.org/gbd-results-tool).^11^ As a signatory of the WHO Guidelines for Accurate and Transparent Health Estimates Reporting, GBD applies standardized data processing to ensure comparability across countries.^12^

By using GHDx, we extracted annual death counts attributed to IHD, stroke, and PAD in Brazil across 5-year age intervals (40-44, 45-49, 50-54, 55-59, 60-64, 65-69, 70-74, and 75-79 years) from 1990 to 2021. The GBD 2021 defines IHD as a composite of acute myocardial infarction, chronic stable angina, coronary artery disease, and ischemic cardiomyopathy, and PAD as an ankle-brachial index < 0.9.^13^ According to the GBD, the original source of mortality data was the Mortality Information System (SIM), a curated and quality-assessed national death certificate database managed by the Brazilian Ministry of Health.^14^ Raw data were processed using the Cause of Death Ensemble model (CODEm), a Bayesian geospatial regression framework developed by the Institute for Health Metrics and Evaluation (Seattle, WA).^13^ Additional details on the GBD 2021 data specific to our study are provided in the supplemental material.

#### Population estimates

We also obtained mid-year population estimates for the same age intervals (40-44 to 75-79 years) for the period 1990 to 2040 from the GBD 2021.^15,16^ The original source of this estimates was the Brazilian Institute of Geography and Statistics, which conducts national censuses in Brazil.^15^ Data were extracted for the overall population, as well as disaggregated by sex.

## Statistical analysis

We first calculated observed annual crude and age-standardized mortality rates (per 100,000 population) for IHD, stroke, and PAD from 1990 to 2021. Age-standardized rates and corresponding 95% confidence intervals (CIs) were computed using the 1990 mid-year population as the standard, with CIs estimated via the delta method.

We then applied Bayesian age-period-cohort (APC) models to the observed data (1990-2021) to project mortality rates for IHD, stroke, and PAD through 2040. APC models allow for simultaneous modeling of age, period, and cohort effects using a single regression equation and can accommodate non-linear relationships.^17^ We used random walk 2 priors with a log-gamma distribution, and modeled the outcome variable (mortality events) using a generalized linear regression framework with a log-link and a Poisson distribution.

Although multiple APC model formulations exist, recent simulation studies have shown that Bayesian APC models offer superior forecasting accuracy.^18^ Estimates from the fitted models were obtained using the integrated nested Laplace approximation, a computationally efficient approach known to produce stable and reliable posterior estimates without convergence issues, comparable to those derived from Markov Chain Monte Carlo simulations.^19^ Detailed information on prior specifications, projection methodology, and model assessment procedures is provided in the supplemental material.

Models were fitted for the total Brazilian population and separately for men and women. For each group, mortality rates were projected for all defined 5-year age intervals, and age-standardized rates were again indexed to the 1990 mid-year population. Projected rates are reported as medians with 95% uncertainty intervals (UIs). We also calculated the relative percentage change and the estimated annual percentage change (EAPC) between the final observed year (2021) and the final projected year (2040). EAPCs were computed using generalized linear regression with a log-link function, and 95% CIs were derived using bootstrap resampling.

All analyses were conducted in R version 4.3.0 (R Foundation for Statistical Computing, Vienna, Austria). As the study relied solely on publicly available, population-level data, it was exempt from Human Research Ethics Committee approval and did not require informed consent. This study was conducted and reported in accordance with the Strengthening the Reporting of Observational Studies in Epidemiology guidelines.

### Patient and public involvement

Patients and members of the public were not involved in the design, conduct, reporting, or dissemination plans of this research, as such involvement was not deemed appropriate or feasible.

## Results

In 1990, Brazil had 32,751,825 residents aged 40-79 years, of whom 51.7% were women. Between 1990 and 2040, this age group is projected to grow by 237.8%, accompanied by substantial population aging (Figure S1). The proportion of adults aged 75-79 years is expected to nearly double, while the share of younger age groups (e.g., 40-44 years) will decline.

The age-standardized IHD mortality rate declined substantially from 1990 to 2021 (Table S1, Figure S2), and this trend is projected to continue through 2040 (Table 1, Figure 1). Although reductions are expected across all age brackets, the greatest decrease is anticipated in individuals aged 60-64 years (Table 1, Figure S3, Figure 2).

**Table 1:** – Projected mortality rates (per 100,000 population) for IHD, stroke, and PAD in Brazil, by 5-year age groups (40-79 years), for 2021 (observed), 2030, and 2040 (projected)

		Year	Age- standardized rate	Age groups (years)							
				40-44	45-49	50-54	55-59	60-64	65-69	70-74	75-79
**IHD**	Observed data	2021	118.61 (117.90-119.32)	21.37 (20.68-22.09)	40.30 (39.27-41.36)	70.76 (69.33-72.23)	112.61 (110.70-114.54)	173.32 (170.74-175.94)	249.85 (246.35-253.40)	354.19 (349.37-359.08)	516.39 (509.22-523.65)
	Projected results	2030	110.76 (72.36-149.16)	19.88 (13.48-29.54)	37.16 (25.90-53.67)	62.84 (44.09-90.21)	99.91 (70.11-143.41)	155.50 (109.13-223.17)	232.25 (162.99-333.31)	347.29 (243.72-498.40)	506.23 (355.24-726.46)
		2040	101.82 (0.36-203.27)	19.04 (6.38-57.74)	35.59 (12.40-103.55)	60.20 (21.46-171.18)	93.99 (33.83-264.76)	141.99 (51.21-399.30)	210.94 (76.06-593.26)	309.77 (111.71-871.19)	453.55 (163.55-1275.56)
	Relative percent change		-14.16%	-10.90%	-11.69%	-14.92%	-16.53%	-18.08%	-15.58%	-12.54%	-12.17%
	EAPC		-0.83 (-0.84a-0.83)	-1.58 (-1.73a-1.43)	-1.63 (-1.76a-1.5)	-1.66 (-1.78a-1.54)	-1.69 (-1.79a-1.58)	-1.7 (-1.79a-1.6)	-1.68 (-1.77a-1.59)	-1.69 (-1.81a-1.59)	-1.72 (-1.86a-1.6)
**Stroke**	Observed data	2021	84.58 (83.99-85.18)	15.24 (14.66-15.85)	27.11 (26.27-27.98)	44.48 (43.35-45.65)	67.22 (65.75-68.72)	106.10 (104.09-108.16)	170.44 (167.56-173.38)	280.48 (276.18-284.83)	467.18 (460.37-474.10)
	Projected results	2030	78.38 (46.18-110.59)	13.56 (8.62-21.37)	23.28 (15.19-35.71)	37.83 (24.84-57.66)	58.81 (38.63-89.63)	94.08 (61.80-143.37)	155.30 (102.02-236.67)	273.05 (179.36-416.09)	466.67 (306.54-711.13)
		2040	69.90 (0-152.48)	12.35 (3.44-44.46)	21.20 (6.13-73.40)	34.45 (10.18-116.68)	52.89 (15.77-177.51)	83.52 (24.96-279.82)	139.11 (41.57-466.15)	240.41 (71.84-805.53)	410.59 (122.69-1375.73)
	Relative percent change		-17.36%	-18.96%	-21.80%	-22.55%	-21.32%	-21.28%	-18.38%	-14.29%	-12.11%
	EAPC		-1.07 (-1.10a-1.04)	-2.77 (-3.02a-2.52)	-2.75 (-2.97a-2.52)	-2.72 (-2.94a-2.51)	-2.67 (-2.87a-2.47)	-2.57 (-2.75a-2.39)	-2.41 (-2.57a-2.26)	-2.27 (-2.42a-2.12)	-2.11 (-2.28a-1.96)
**PAD**	Observed data	2021	1.82 (1.73-1.91)	0.13 (0.09-0.20)	0.27 (0.20-0.38)	0.58 (0.46-0.73)	1.28 (1.09-1.50)	2.38 (2.10-2.71)	4.05 (3.62-4.53)	7.12 (6.46-7.85)	12.08 (11.03-13.24)
	Projected results	2030	2.01 (0.91, 3.10)	0.12 (0.06-0.24)	0.26 (0.14-0.48)	0.56 (0.31-0.98)	1.17 (0.64-2.01)	2.34 (1.29-4.00)	4.52 (2.50-7.72)	8.44 (4.68-14.42)	14.92 (8.27-25.49)
		2040	2.02 (0-5.03)	0.12 (0.02-0.63)	0.26 (0.05-1.24)	0.55 (0.11-2.51)	1.14 (0.23-5.06)	2.28 (0.47-9.94)	4.41 (0.90-19.10)	8.49 (1.74-36.74)	16.23 (3.33-70.24)
	Relative percent change		+10.99%	-7.69%	-3.70%	-5.17%	-10.94%	-4.20%	+8.89%	+19.24%	+34.35%
	EAPC		0.45 (0.30-0.59)	-0.21 (-0.22a-0.21)	-0.21 (-0.21a-0.21)	-0.21 (-0.21a-0.21)	-0.23 (-0.24a-0.23)	-0.21 (-0.24a-0.21)	0.07 (-0.08-0.07)	0.70 (0.45-0.70)	1.4 (1.17-1.40)

Age-standardized and age-specific rates are presented as medians with 95% uncertainty interval for 2030 and 2040 projections, and as crude rates with 95% CIs for the observed year (2021). Relative percentage change and EAPC between 2021 and 2040 are also provided. CI: confidence interval; EAPC: estimated annual percent change; IHD: ischemic heart disease; PAD: peripheral artery disease.

**Figure 1:** – Projected age-standardized mortality rates in Brazil through 2040. Panels A, B, and C depict age-standardized mortality rates (per 100,000 population) for IHD, stroke, and PAD, respectively. Estimates were obtained using Bayesian age-period-cohort models based on observed data from 1990 to 2021. Black dots represent observed values (1990-2021), while the fan plots illustrate projected estimates for 2022-2040. Shaded regions indicate uncertainty intervals: the darker bands represent the interquartile range (25th to 75th percentile), and the lighter bands represent the 95% UI (2.5th to 97.5th percentile). IHD: ischemic heart disease; PAD: peripheral artery disease; UI: uncertainty interval.

**Figure 2:** – Projected change in IHD, stroke, and PAD mortality in Brazil by age group, 2021-2040. This figure shows the EAPC in mortality rates (per 100,000 population) for IHD, stroke, and PAD across 5-year age brackets in Brazil, projected from 2021 to 2040. Bars represent point estimates, and error bars indicate 95% CIs. Full EAPC values by age group and condition are presented in Table 2.

EAPC: estimated annual percentage change; IHD: ischemic heart disease; PAD: peripheral artery disease.

Between 1990 and 2021, age-standardized IHD mortality declined for both men and women, although rates consistently remained higher in men (Table S1). Between 2021 and 2040, the age-standardized IHD mortality rate is projected to decrease by 25.33% in men but increase by 4.13% in women (Table S2, Figure 3, and Figure 4). As shown in Figure 4, the gap in IHD mortality between men and women is expected to narrow over time.

**Figure 3:** – Projected change in ischemic heart disease (IHD), stroke, and peripheral artery disease (PAD) mortality in Brazil through 2040. This figure presents the estimated annual percentage change (EAPC) in age-standardized mortality rates (per 100,000 population) for IHD (Panel A), stroke (Panel B), and PAD (Panel C) between 2021 and 2040. Results are shown for the overall population, as well as disaggregated by sex (men and women).

**Figure 4:** – Projected age-standardized mortality rates in men and women in Brazil through 2040. Panels A, B, and C show age-standardized mortality rates (per 100,000 population) for ischemic heart disease, stroke, and peripheral artery disease, respectively, stratified by sex — men (red) and women (blue). Dots represent observed data from 1990 to 2021, while lines correspond to projections for 2022-2040 based on Bayesian age-period-cohort models. Solid lines indicate median estimates; dashed lines represent the interquartile range (25th to 75th percentile); and dotted lines reflect the 95% UI (2.5th to 97.5th percentile). UI: uncertainty. interval.

Similarly, age-standardized stroke mortality declined substantially from 1990 to 2021 (Table S3, Figure S2). This downward trend is projected to persist through 2040, with an overall reduction of 17.36% in Brazil (Table 1, Figure 1). Among age groups, the greatest decline is expected in individuals aged 50-54 years (Table 1, Figure S4, Figure 2).

From 1990 to 2021, stroke mortality rates decreased in both sexes (Table S3). Between 2021 and 2040, further reductions are projected; however, the decline will be markedly greater among men (30.02%) compared to women (4.51%) (Table S4, Figure 3 and Figure 4). As illustrated in Figure 4, the sex gap in stroke mortality is also expected to diminish over time.

Regarding PAD, the age-standardized mortality rate initially declined between 1990 and 2015, followed by a sharp increase up to 2021 (Table S5, Figure S2). Between 2021 and 2040, PAD mortality in Brazil is projected to rise by 10.99% (Table 1, Figure 1). Notably, the projected trends vary substantially by age. While a 7.69% decline is anticipated in the 40-44 age group, mortality is expected to increase by 34.35% in those aged 75-79 years (Table 1, Figure S5, Figure 2).

The trend of an initial decline followed by a subsequent sharp increase in PAD mortality was observed in both men and women. Throughout the observed period, men consistently exhibited higher PAD mortality rates (Table S5). Between 2021 and 2040, age-standardized PAD mortality is projected to increase by 14.61% in men and 21.99% in women (Table S6, Figure 3 and Figure 4).

The Central Illustration provides a graphical summary of the study’s key findings and methodological approach.

## Discussion

We examined the observed burden of IHD, stroke, and PAD mortality among Brazilian adults from 1990 to 2021 and fitted models to project future estimates through 2040. Our findings indicate that age-standardized mortality rates for IHD and stroke have steadily declined over the past 3 decades and are projected to continue decreasing. In contrast, PAD mortality is expected to rise between 2021 and 2040, with a disproportionately greater increase among women.

The observed decline in IHD and stroke mortality since 1990 aligns with prior findings reported by Bastos et al., who noted similar trends but highlighted that improvements were attenuated in socioeconomically vulnerable regions, particularly in North and Northeast Brazil.^20^ The Brazilian Society of Cardiology (SBC) has also reported continued reductions in IHD and stroke mortality, despite the adverse impact of COVID-19 on cardiovascular outcomes.^3,21^ Comparable projections have been published for countries such as the United States, United Kingdom, and Japan.^9,10,22^ Together with our findings, this body of evidence is encouraging, as it suggests that IHD and stroke mortality may continue to decline despite significant population aging.

However, our study also revealed that the projected decline in stroke mortality will be considerably smaller among older adults. A previous study analyzing U.S. data reported minimal reductions in stroke mortality among individuals aged 65-74, 75-84, and 85 years or older for both sexes.^9^ These findings underscore the need for targeted stroke prevention and care strategies in older populations. Importantly, IHD and stroke share several modifiable risk factors. A recent meta-analysis involving 50,000 patients showed that approximately one-third of acute stroke patients also have IHD and are at increased risk of myocardial infarction within 1 year following a stroke.^23^

In Brazil, effective acute stroke care is challenged by large rural populations and unequal distribution of health care resources. Although dedicated stroke units and fibrinolysis programs have been implemented in several metropolitan centers, access remains limited in rural and underserved regions.^24^ While existing health care policies have contributed to improvements in IHD and stroke mortality, further efforts are necessary to consolidate these gains — especially in view of Brazil’s rapidly aging population.

Our study also highlights 2 particularly concerning issues: (i) the growing burden of PAD mortality in Brazil, and (ii) significant sex differences in the projected mortality rates for IHD, stroke, and PAD. According to the GBD, the global burden of PAD increased by 72% between 1990 and 2019.^25^ Projections by the Institute for Health Metrics and Evaluation — developers of the GBD — indicate that low- and middle-income countries will bear a disproportionately greater share of this burden.^2^ Advanced PAD is associated with high rates of lower limb amputation, poor quality of life, substantial morbidity, and elevated 5- to 10-year mortality.^27,28^ These outcomes underscore the urgency of implementing public policies focused on early PAD detection and management.

The second key issue identified is the marked disparity in cardiovascular outcomes between sexes. A study from Spain found that, compared to men, women experienced longer delays in the diagnosis and treatment of acute myocardial infarction.^28^ In the context of PAD, vascular surgery outcomes are also poorer for women, who are more likely to present with acute limb ischemia.^26,29^ According to the 2023 report from the Brazilian Ministry of Health and the SBC, obesity rates are higher among women than men, and women are 44% more likely to have dyslipidemia.^3^ Higher PAD prevalence and mortality in women may also serve as a surrogate for undiagnosed or untreated atherosclerosis, which is known to be more prevalent in women. Taken together, these findings emphasize the urgent need for targeted health initiatives aimed at addressing the observed sex disparities in cardiovascular outcomes.

In 2011, Brazil’s Ministry of Health launched a 10-year strategic plan to reduce non-communicable disease rates. Since then, the country has achieved important gains, including a 30% reduction in smoking prevalence and a 10% increase in physical activity among adults.^30^ These are significant achievements, considering that approximately 70% of the total burden of CVD is attributable to modifiable risk factors.^2^ However, as observed in many other countries, Brazil continues to experience rising rates of adult obesity as well as increased prevalence of hypertension, diabetes, and dyslipidemia.^30^

In response, the Brazilian government — alongside the SBC and other medical associations — introduced a new national CVD plan in 2023, which is currently under review by the Senate.^31^ This proposed legislation seeks to address current challenges by implementing targeted interventions and establishing a dedicated federal fund to combat CVD. The proposed allocation of this fund includes: 30% for CVD-related research; 15% for public awareness campaigns on cardiovascular risk factors; 30% for training health professionals, particularly in primary care; and 15% for programs aimed at reducing smoking, alcohol consumption, and other modifiable risk factors. Additionally, 10% of the fund would be managed by the Brazilian Federal Council of Medicine to support specialized training in cardiovascular care. Such initiatives could help reverse the recent trends of underinvestment in Brazil’s public health care system.^32^

Improving medication adherence and expanding access to care — especially by increasing the number and geographic distribution of health professionals trained in preventive cardiology — are also critical strategies for effectively addressing the national CVD burden.^5,33,34^

Beyond traditional cardiovascular risk factors, it is essential to recognize the role of environmental exposures, such as air pollution from fine particulate matter, which has been causally linked to CVD. Encouragingly, recent data show that air pollution-related mortality in Brazil declined between 2010 and 2019.^35^ However, the same study reported that pollution levels in certain regions remained multiple times higher than the limits recommended by the WHO.^35^ Thus, future policy efforts should not only continue to address conventional CVD risk factors but also expand to include environmental determinants of cardiovascular health.

Finally, as increasingly discussed within the cardiology community, the development of preventive cardiology guidelines specifically tailored to women is a crucial step toward reducing the sex-based disparities identified in our study.^36-39^

### Strengths and limitations

This study has several limitations that should be acknowledged. Both event rates and mid-year population estimates were obtained from the GBD 2021 dataset. While these data are originally sourced from the SIM, the GBD applies its own modeling procedures. As a result, our findings may differ from studies that directly abstract data from the original source.^40,41^ Nevertheless, because the GBD harmonizes data across countries, our results are more readily comparable to international studies.

Although the SIM routinely conducts quality checks to ensure accuracy, death certification remains the primary source of mortality information. The accuracy of these records may vary, particularly in resource-limited rural regions. However, the GBD methodology is specifically designed to address issues of data completeness and misclassification.

Our study does not account for individual-level or population-level risk factors. Future trends in IHD, stroke, and PAD mortality may be significantly influenced by changes in these factors. While more aggressive mitigation strategies could reduce future event rates, current trends — such as rising prevalence of obesity, diabetes, and tobacco use — could potentially lead to even higher mortality than projected.

We employed Bayesian APC models to project future mortality estimates. Like all modeling approaches, these require certain assumptions. Although model misspecification remains a possibility, we reported 95% UIs to account for such limitations. Furthermore, public health outcomes are influenced by a broad array of factors — including political, cultural, migratory, and environmental changes — many of which cannot be fully captured by the mathematical models used in this study.

## Conclusion

Based on observed data from 1990 to 2021, age-standardized mortality from IHD and stroke in Brazil is projected to continue declining through 2040. In contrast, age-standardized mortality attributable to PAD is expected to rise substantially over the same period. While the projected reductions in IHD and stroke mortality will be more pronounced among men, the increase in PAD mortality is anticipated to be considerably higher among women. These findings underscore the urgent need for sustained and targeted public health interventions to reduce cardiovascular health disparities, increase awareness, and improve equitable access to evidence-based care across Brazil.

## *Supplemental Materials

For additional information, please click here. https://abccardiol.org/supplementary-material/2025/12212/2025-0189_final_supplemental_section.pdf
